# Changes in the spike and nucleocapsid protein of porcine epidemic diarrhea virus strain in Vietnam—a molecular potential for the vaccine development?

**DOI:** 10.7717/peerj.12329

**Published:** 2021-10-18

**Authors:** Thach Xuan Tran, Nguyen T.K. Lien, Ha T. Thu, Nguyen Dinh Duy, Bui T.T. Duong, Dong Van Quyen

**Affiliations:** 1 Dept of Molecular Microbiology, Institute of Biotechnology, Hanoi, Vietnam; 2 Functional of Genomics Lab, Institute of Genome Research, Vietnam Academy of Science and Technology, Hanoi, Vietnam; 3 University of Science and Technology of Ha Noi, Vietnam Academy of Science and Technology, Hanoi, Vietnam

**Keywords:** Porcine epidemic diarrhea virus, Spike protein, Neutralizing antibodies, Phylogenetic analysis, Pigs

## Abstract

**Background:**

Porcine epidemic diarrhea virus (PEDV) is a dangerous virus causing large piglet losses. PEDV spread rapidly between pig farms and caused the death of up to 90% of infected piglets. Current vaccines are only partially effective in providing immunity to suckling due to the rapid dissemination and ongoing evolution of PEDV.

**Methods:**

In this study, the complete genome of a PEDV strain in Vietnam 2018 (IBT/VN/2018 strain) has been sequenced. The nucleotide sequence of each fragment was assembled to build a continuous complete sequence using the DNASTAR program. The complete nucleotide sequences and amino acid sequences of S, N, and ORF3 genes were aligned and analyzed to detect the mutations.

**Results:**

The full-length genome was determined with 28,031 nucleotides in length which consisted of the 5′UTR, ORF1ab, S protein, ORF3, E protein, M protein, N protein, and 3′UTR region. The phylogenetic analysis showed that the IBT/VN/2018 strain was highly virulent belonged to the G2b subgroup along with the Northern American and Asian S-INDEL strains. Multiple sequence alignment of deduced amino acids revealed numerous mutations in the S, N, and ORF3 regions including one substitution ^766^P > L^766^ in the epitope SS6; two in the S^0^subdomain (^135^DN^136^ > ^135^SI^136^ and N^144^> D^144^); two in subdomain S^HR1^ at aa ^1009^L > M^1009^ and ^1089^S > L^1089^; one at aa ^1279^P > S^1279^ in subdomain S^HR2^ of the S protein; two at aa ^364^N > I^364^ and ^378^N > S^378^ in the N protein; four at aa ^25^L > S^25^, ^70^I > V^70^, ^107^C > F^107^, and ^168^D > N^168^ in the ORF3 protein. We identified two insertions (at aa ^59^NQGV^62^ and aa ^145^N) and one deletion (at aa ^168^DI^169^) in S protein. Remarkable, eight amino acid substitutions (^294^I > M^294^, ^318^A > S^318^, ^335^V > I^335^, ^361^A > T^361^, ^497^R > T^497^, ^501^SH^502^ > ^501^IY^502^, ^506^I > T^506^, ^682^V > I^682^, and ^777^P > L^777^) were found in S^A^ subdomain. Besides, N- and O-glycosylation analysis of S, N, and ORF3 protein reveals three known sites (25^G+^, 123^N+^, and 62^V+^) and three novel sites (144^D+^, 1009^M+^, and 1279^L+^) in the IBT/VN/2018 strain compared with the vaccine strains. Taken together, the results showed that mutations in the S, N, and ORF3 genes can affect receptor specificity, viral pathogenicity, and the ability to evade the host immune system of the IBT/VN/2018 strain. Our results highlight the importance of molecular characterization of field strains of PEDV for the development of an effective vaccine to control PEDV infections in Vietnam.

## Introduction

Porcine epidemic diarrhea (PED) characterized by watery diarrhea, vomiting, and severe dehydration in suckling piglets and led to 50%–90% mortality among susceptible piglets (Madson et al., 2014). The disease is caused by the porcine epidemic diarrhea virus (PEDV), an enveloped, positive-sense single-stranded RNA virus that belongs to the *Coronaviridae* family in Nidovirales order (genus *Alphacoronavirus*) (Lin et al., 2016)*.* The genome of PEDV is approximately 28 kb in length. It comprises a 5′ untranslated region (5′ UTR), a 3′ UTR with a polyadenylated tail, and seven open reading frames (ORF1a, ORF1b, and ORF2-6) encoding four structural proteins (spike, S; envelope, E; membrane, M; nucleocapsid, N), two nonstructural proteins, and one accessory protein ORF3 (Song & Park, 2012).

The S protein (a glycoprotein) contains a specific receptor binding site that is an antigenic target for neutralizing antibodies and relates to its pathogenicity and immunogenicity (Pospischil, Stuedli & Kiupel, 2002). This protein plays a critical role in viral entry through the viral-cellular fusion activity and induces an immune response in the natural host during replication (Lee, 2015; Teenavechyan et al., 2016). Thus, it is used to study the genetic match between vaccine strains and circulating PEDV strains (Lee et al., 2010; Lee & Lee, 2014; Oh et al., 2014). The M protein is a surface protein and plays an important role in the process of virus-assembly and the induction of protective antibodies with neutralizing activity (Lee, 2015; Teenavechyan et al., 2016). The N protein is highly conserved and binds to virion RNA to provide a structural basis for viral transcription, replication, and assembly (Curtis, Yount & Baric, 2002; Chen et al., 2013a; Sun et al., 2015). The N protein was commonly used to diagnose infection with PEDV (Song, Moon & Kang, 2015a) and was known to protect the viral genome during the coronavirus assembly. It also affects other anti-virus responses through host immune evasion strategies (Lee, 2015; Teenavechyan et al., 2016). The epitopes on the N protein are considered to possibly cause for induction of cell-mediated immunity (CMI) (Saif, 1993). However, protein S is more antigenic than other PEDV proteins and anti-S antibodies detected in PEDV-infected pigs last longer than anti-N antibodies (Knuchel et al., 1992). For non-structural proteins, ORF1a and ORF1b are multifunctional related to viral genome replication (Brian & Baric, 2005), and the accessory ORF3 protein is known to be associated with the virus virulence (Park et al., 2007; Park et al., 2008; Wang et al., 2012; Chen et al., 2013b; Song et al., 2015b). Therefore, the ORF3 protein has been studied extensively in the molecular epidemiology of PEDV (Shirato et al., 2011; Chen et al., 2013a; Li et al., 2013; Temeeyasen et al., 2014; Song et al., 2015b). These proteins have been the targets of many studies to understand the causes of the outbreaks and develop more effective vaccines (Lee & Lee, 2014; Temeeyasen et al., 2014; Lee, 2015; Song et al., 2015b; Lin et al., 2016; Su et al., 2016).

Won et al. (2020) reviewed 299 published articles and showed that current vaccines are produced mainly based on PEDV strains: CV777 (belonged to G1a group) and SM98, DR13 (belonged to G2a group). The G2b whole-virus killed vaccines have been developed and used in the USA and the G2b live oral vaccine (based on the KNU-141113 strain) has been developed and used in Korea from 2020. However, the virus evoluted and accumulated mutations as the time passed that may lead to sub-optimally match to the actual pandemic virus of the vaccines. Therefore, complete genome sequencing of PEDV strains circulating in each country is of critical in order to develop effective vaccines (Vlasova et al., 2014; Vui et al., 2015; Jarvis et al., 2016; Fan et al., 2017; Lee & Lee, 2017; Rasmussen et al., 2018; Liang et al., 2020; Garcia-Hernandez et al., 2021; Wen et al., 2021).

In Vietnam, PEDV caused an outbreak for the first time in 2009 and then occurred again in 2013 (Diep et al., 2018). Several PEDV vaccines have been developed and being used in Vietnam based on vaccine strains CV777/CN/KT323979, SM98/Korea/GU937797, and DR13/Korea/JQ023162 (belonged to G1a and G2a groups). Although a vaccine *campaign* for piglets has been conducted since 2011 for PEDV control, diarrhea disease still exists in many provinces and causes serious damage to the livestock industry in recent years in Vietnam. The low effectiveness of the vaccines could be due to the genetic differences between vaccines and field epidemic strains, emphasizing the necessity of novel vaccines against new viral strains. Vui et al. (2015) has sequenced the first complete genome of PEDV from outbreaks in Vietnam in 2013 and suggested that the Vietnamese PEDV isolates were new variants. Other studies mainly focused on the spike gene sequences indicated that there have been remarkable changes in the Vietnamese PEDV strains collected from 2012 to 2016 (Kim et al., 2015; Diep et al., 2018; Than et al., 2020) and these may decrease the vaccine efficiencies. Although PEDV strains remain the main cause of piglet losses in large litters in Vietnam; however, very limited molecular data of their genotypic and genealogy of PEDV are available from Vietnam. Since the first complete genome sequence of Vietnamese PEDV strains announced in 2015, no more complete sequence of Vietnamese PEDV strain was reported. In this study, we report the molecular characteristics and the changes in the spike and nucleocapsid protein of a PEDV strain isolated from pigs causing severe diarrhea in 2018 in Hung Yen province, Northern Vietnam. Our study emphasizes the importance and urgency of studying the genetic diversity of current PEDV strains and their evolution in order to develop a suitable vaccine strategy for each geographic region.

## Materials and Methods

### Sample collection and RNA extraction

The PED-specific PCR-positive fecal samples were collected from the piglets that died of diarrhea in 2018, in Hung Yen province (in North of Vietnam), transported to the laboratory, and stored at −20 °C until used. Total RNA was extracted using Trizol reagent (Merck, Darmstadt, Germany) according to the manufacturer’s instructions and suspended in DEPC-treated water (Thermo Fisher Scientific Inc, Waltham, MA, USA) and stored at −80 °C until use.

### cDNA synthesis and genome sequencing

The first-strand cDNA synthesis was performed using the Maxima Reverse Transcriptase Kit (Thermo Fisher Scientific Inc, Waltham, MA, USA) following the manufacturer’s protocols. The complete genome sequence of the PEDV strain was amplified by 20 pairs of primers (Table 1) designed based on the conserved regions of the reference strains of PEDV available on GenBank. Each DNA fragment was amplified by using PCR master mix (2X) (Thermo Fisher Scientific Inc, Waltham, MA, USA), and the thermal cycle was performed at 94 °C for 3 min, followed by 35 cycles of 94 °C for 1 min, 50 °C to 56 °C for 40 s, and 72 °C for 1 min, with a final extension at 72 °C for 8 min. The PCR products were purified from agarose gel using GeneJet Gel Extraction Kit (Thermo Fisher Scientific Inc, Waltham, MA, USA) and subjected to DNA sequencing by using an automated sequencer (ABI 3500 Genetic Analyzer). The nucleotide sequence of each fragment was assembled to build a continuous complete sequence using the DNASTAR program.

**Table 1:** Sequences of primer pairs used for amplification of the whole genome of the IBT/VN/2018 strain.

Primer	Sequence (3′–5′)	Position on the genome	%GC	Tm (°C)	Length (bp)
PEDV_1F	ACTTAAARAGATTTTCTATCTACG	1–24	25	47	1,625
PEDV_1R	TTAACGATACTAAGAGTGGC	1606–1625	40	48	
PEDV_2F	GCTGGTCATGTTGTTGTTG	1423–1441	49	49	1,783
PEDV_2R	TAGATGTAGTACTTAGGCAC	3187–3206	40	48	
PEDV_3F	TTCTCTGATGAAGTCTCTG	2905–3013	42	47	1,871
PEDV_3R	CATGAGCACCTTCCAATCCTG	4755–4776	50	51	
PEDV_4F	TTGCATGTGTTGGTGATCGC	4568–4587	50	50	1,580
PEDV_4R	CCGATGCATAATTCATAGTGTC	6127–6148	41	51	
PEDV_5F	GATCATGGCACTGGTATGGTGC	5974–5995	52	54	1,675
PEDV_5R	TCTTGGCACCTACACGCATAC	7628–7648	52	54	
PEDV_6F	CAGGATTGCAAGAGCACATTG	7456–7476	48	52	1,678
PEDV_6R	AGTACTAGCATACTGACGCAG	9112–9132	48	52	
PEDV_7F	CTYATTGCACCATGGTGGG	8908–8926	53	51	1,710
PEDV_7R	AGCTACCACATAAGTGACAG	10598–10617	45	49	
PEDV_8F	GCTCTGATTGTTACATCTTGC	10405–10425	43	50.5	1,621
PEDV_8R	CACTTAACTACACGCAGGTC	12007–12026	50	51	
PEDV_9F	ATGCTGAGTCCCTGTCATG	11810–11828	53	51	1,617
PEDV_9R	AGTGCTGTCTTATGCTCCGTG	13406–13426	52	54	
PEDV_10F	AGGTATAGTTGGTGTTGTCAC	13175–13195	43	50	1,636
PEDV_10R	AACGACGAACTGGTCATCGAC	14790–14810	52	54	
PEDV_11F	ACCTCTGGTGATGCAACCAC	14622–14641	55	54	1,670
PEDV_11R	ATACGTGCACCTGGATAGTAC	16271–16291	48	52	
PEDV_12F	TACCTCACATAATGTTCAGCC	16061–16081	43	50	1,649
PEDV_12R	TAGTATGTCTGATAGGTTRGCC	17690–17710	41	51	
PEDV_13F	TGTTGTCAGACCTGAAGGTTG	17519–17540	48	52	1,615
PEDV_13R	TGTAAGTGACATAAGCACAGC	19113–19133	43	50	
PEDV_14F	AGCGTAAGGTAGGACTCAC	18898–18916	53	51	1,617
PEDV_14R	CAACTGTAGCCTTATGCTTAC	20494–20514	43	50.5	
PEDV_15F	TGGACAATGTTCTGTACCAG	20301–20322	45	50	1,649
PEDV_15R	AACRTCATCGTCAGTGCCATG	21929–21949	52	54	
PEDV_16F	CATACTGCTYTAGGAACAAAYC	21656–21677	40	50	1,664
PEDV_16R	TGTACCACCCTGCCACTTGC	23300–23319	60	55	
PEDV_17F	GACCATAGAGTCAGCATTACAAC	23137–23159	43	53.5	1,640
PEDV_17R	TCGTAAGGTTGAAGTCTAGGAC	24756–24777	45	53	
PEDV_18F	AAGTGGCCGTGGTGGGTTTG	24617–24636	60	56	1,606
PEDV_18R	AGTGGCCTTGGCGACTGTGAC	26201–26222	62	58	
PEDV_19F	TAGCATTCGGTTGTGGCGC	25991–26009	58	53	1,511
PEDV_19R	CACCTGTGAAACAAGAAGCTC	27482–27502	48	52	
PEDV_20F	AGTGGAGGAGAATTCCCAAG	27228–27247	50	51	820
PEDV_20R	TGTATCCATATCAACACCGTC	28026–28048	43	51	

### Phylogenetic analysis

Phylogenetic trees were constructed based on the full-length genome and S nucleotide sequences by the neighbor-joining method using MEGA 7 software (Kumar, Stecher & Tamura, 2016) of the Vietnamese strain with 72 reference strains (Table 2) archived from GenBank including the vaccine strains (CV777/CN/KT323979, AJ1102/CN/JX188454, SM98/Korea/GU937797, and DR13/Korea/JQ023162) and the representative strains from Asia (China, Korea, Japan, Thailand, and Taiwan), Europe (Belgium, France, and German), and America (USA and Mexico). The bootstrap values were calculated based on 1,000 replicates.

**Table 2:** Representative PEDV strains used in this study.

**Strain**	**Country**	**Year**	**ACNO**	**Strain**	**Country**	**Year**	**ACNO**
CV777	Belgium	2001	AF353511	DR13	Korea	2009	JQ023161
15V010/BEL	Belgium	2015	KR003452	S protein	Korea	2006	DQ862099
L00721	GER	2014	LM645057	S protein	Korea	2002	AF500215
L01330	GER	2015	LT898435	S protein	Korea	2009	GU180144
Br1-87	GER	2018	LT906582	KNU-0801	Korea	2008	GU180142
FR001	France	2014	KR011756	CNU091222	Korea	2008	JN184635
MEX124	Mexico	2014	KJ645700	SM98	Korea	2010	GU937797
Indiana1283	USA	2013	KJ645704	AD01	Korea	2011	KC879280
Minnesota52	USA	2013	KJ645704	AD02	Korea	2012	KC879281
MN	USA	2013	KF468752	KDJN12YG	Korea	2012	KJ857475
IA1	USA	2013	KF468753	KNU-1302	Korea	2013	KJ451037
IA2	USA	2013	KF468754	KNU-1310	Korea	2013	KJ451045
PC22A-P3	USA	2013	KU893861	KNU-1305	Korea	2013	KJ662670
Ohio126	USA	2014	KJ645702	KNU-1401	Korea	2014	KJ451047
OH851	USA	2014	KJ399978	KNU1406-1	Korea	2014	KM403155
Minnesota271	USA	2014	KR265813	KNU141112	Korea	2014	KR873431
KH	JPN	2011	AB548622	KNU-1601	Korea	2016	KY963963
NK	JPN	2011	AB548623	TW-63	Taiwan	2014	KP276250
KGS-1	JPN	2013	LC063814	JS2004	CN	2004	AY653204
OKN-1	JPN	2013	LC063836	LJB03	CN	2006	DQ985739
IWT-4	JPN	2014	LC063813	JS2008	CN	2008	KC109141
KCH-2	JPN	2014	LC063845	HBQX10	CN	2010	JX501318
NPPED0108-1	Thailand	2008	KC764953	JLCC	CN	2011	JQ638920
NPPED0108-2	Thailand	2008	KC764952	BJ2011-1	CN	2011	JN825712
PED0210-2	Thailand	2010	KC764955	GD1	CN	2011	JX647847
SBPED0211-3	Thailand	2011	KC764959	GDA	CN	2012	JX112709
SPPED0212-1	Thailand	2012	KC764958	SDM	CN	2012	JX560761
6-56ST0413	Thailand	2013	KF724938	YNKM-8	CN	2013	KF761675
CBR1	Thailand	2014	KR610993	YC2014	CN	2014	KU252649
HUA PED45	VN	2013	KP455313	SXYL	CN	2016	MF462814
HUA PED47	VN	2013	KP455314	CH hubei	CN	2016	KY928065
HUA PED67	VN	2013	KP455319	JXJA	CN	2017	MF375374
HUA PED96	VN	2013	KT941120	VAP	VN	2013	KJ960178
KCHY	VN	2013	KJ960180	JFP	VN	2013	KJ960179

**Notes.**

ACNO Accession Number GER Germany USA United States of America JPN Japan VN Vietnam CN China

### Multiple sequence alignments of S, N and ORF3 genes

The complete nucleotide sequences and amino acid sequences of S, N, and ORF3 genes were aligned and compared by the BioEdit software (version 7.0.9.0) (Hall, 1999) to detect the mutations.

### *In silico* glycosylation and homology modeling analysis

The glycosylation analysis was performed to detect the glycosylation sites in the S, N, and ORF3 proteins using the glycosylation prediction software (Hamby & Hirst, 2008). For homology modeling analysis, the S protein sequence of the Vietnamese strain was compared with the S protein sequence ID 6U7K in PDB by SPDV software (Guex & Peitsch, 1997).

## Results

### Genome analysis

The complete genome of the IBT/VN/2018 strain was determined and deposited in GenBank under accession number MT198679. The full-length genome was 28,031 nucleotide (nt) in length, excluding the 3′ poly-A. It consisted of a 5′ UTR region with 292 nt in length; an ORF1ab region with 20,248 nt; a S protein with 4,158 nt (1,385 aa); an ORF3 region with 675 nt (224 aa); an E protein with 231 nt (76 aa); a M protein with 681 nt (226 aa); a N protein with 1,326 nt (441 aa); and a 3′ UTR region with 229 nt. Full-length genome analysis indicated that IBT/VN/2018 strain shared nucleotide similarity ranged from 95.7%–99.3% in comparison with other PEDV strains (Table S1). The lowest genetic identity was 95.7% compared with the CBR1 strain (KR610993) isolated from Thailand in 2014 and the highest identity was 99.3% compared with the CH Hubei strain (KY928065) isolated from China in 2016.

### Phylogenetic analysis

**Figure 1:**
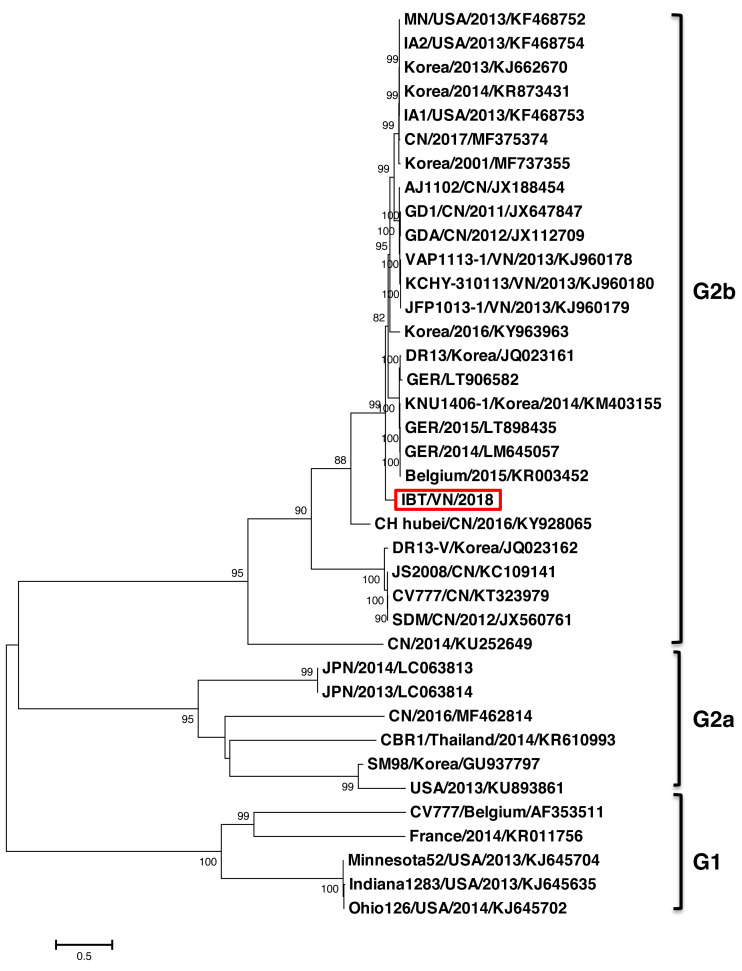
Phylogenetic analysis of the complete nucleotide sequences of PEDV strains. Phylogenetic tree was constructed base on the Northern of Vietnam (IBT/VN/2018) strain, the vaccine strains, and those of other reference strains (USA, France, Germany, Belgium, China, Korea, Japan, Thailand, Vietnam). Multiple alignment was performed using MEGA7 software with 1,000 replicates bootstrap values.

The phylogenetic tree constructed based on the full-length genome sequence of 37 PEDV references indicated that the PEDV strains could be divided into two major groups G1 and G2 (Fig. 1). Group 1 contained five strains including CV777 (Belgium/2001/AF353511), FR001 (France/2014/KR011756), Minnesota52 (USA/2013/KJ645704), and Ohio126 (USA/2014/KJ645702). Group 2 was divided into two subgroups G2a and G2b. The G2a group comprised of six strains from countries including Japan (JPN/2014/LC063813, JPN/2013/LC063814), Korea (SM98/2010/GU937797), China (SXYl/2016/MF462814), Thailand (CBR1/2014/KR610993), and USA (PC22A/2013/KU893861). The G2b group, which is considered highly virulent, comprised of strains from Vietnam (IBT/VN/2018, KCHY, VAP, and JFP), China, Korea, USA, Germany, and Belgium. Remarkably, the PEDV strain isolated from North of Vietnam and CH Hubei/CN/2016/KY928065 strain—a highly pathogenic strain from China in 2016, showed the highest genetic identity with each other compared to other strains.

**Figure 2:**
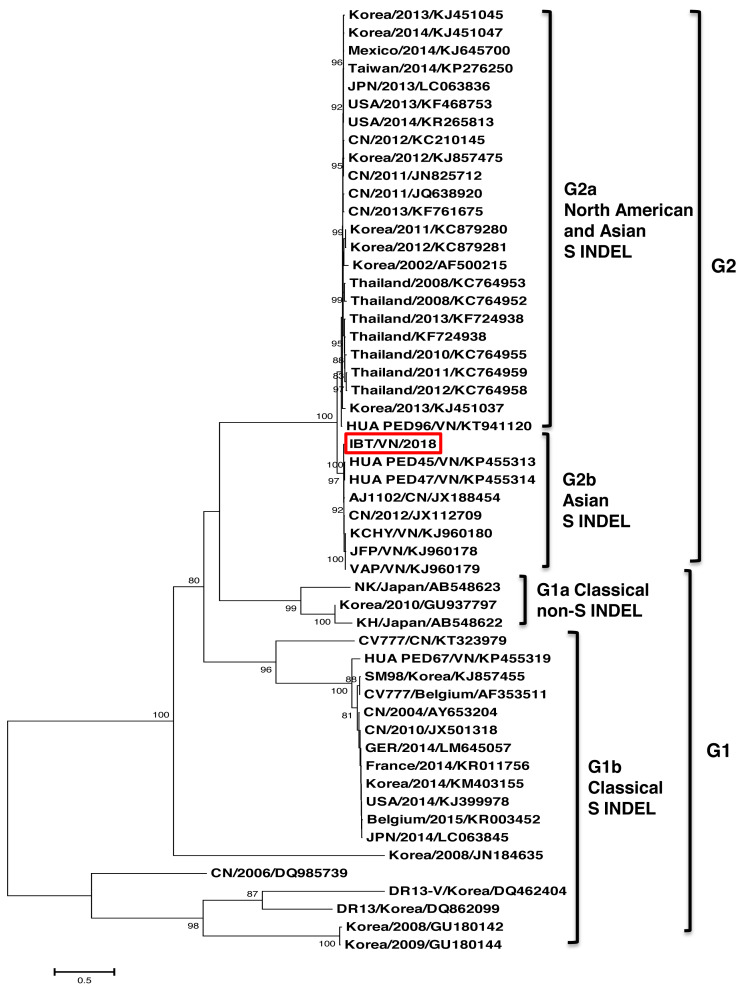
Phylogenetic relationships based on nucleotide sequences of the spike protein. The phylogenetic tree was constructed using the neighbor-joining method in the MEGA7 software (with 1,000 replicates bootstrap values).

The phylogenetic tree based on the S protein sequences also showed that all the strains could be divided into two groups (Fig. 2). Group 1 (G1) consisted of two subgroups: G1a and G1b. G1a subgroup included the classical non S-INDEL strains (Korea/2010/GU937797, JPN/2011/AB548622, and JPN/2011/AB548623). G1b subgroup included the classical S-INDEL strains (Belgium/2015/KR003452, CV777/Belgium/AF353511, CV777/CN/KT323979, SM98/Korea/KJ857455, DR13-V/Korea/DQ462404, DR13/Korea/DQ862099, HUA PED67/VN/KP455319, and some strains from France, Germany, USA, China, Japan, and Korea). Group 2 (G2) contained two subgroups: G2a and G2b. Among them, the Vietnamese strains (IBT/VN/2018, HUA PED45, HUA PED47, KCHY, VAP, and JFP) belonged to the G2b subgroup including Asian S-INDEL strains such as the vaccine strain AJ1102/CN/JX188454 and China/2012/JX112709. G2a subgroup including 24 strains from USA, Mexico, Korea, China, Taiwan, and Thailand belonged to Northern American and Asian S-INDEL subgroup.

### Multiple sequence alignments of S, N and ORF3 genes

The S gene sequence of the IBT/VN/2018 strain was 4,158 nt in length and encoded a protein consisting of 1,385 aa. The genetic identity of the nucleotide sequence and amino acid sequence of the S gene of the Vietnamese strain was 92.2%–98.1% and 91.1%–98.4% compared to the strains from other countries, respectively (Table S2). The highest identity was found between IBT/VN/2018 strain and CN/2012/JX112709 strain, while the lowest identity was found with Korea/2010/GU937797 strain. The S protein of the IBT/VN/2018 strain contained a signal peptide (aa 1–19), four neutralizing epitopes (aa 501–640, 751–758, 766–774, and 1,370–1,376), a transmembrane domain (aa 1,328–1,350), and a short cytoplasmic domain. Four epitopes were determined at aa positions 501–640 (COE), ^751^YSNIGVCK^758^ (SS2), ^766^LSQSGQVKI^774^(SS6), and ^1370^GPRLQPY^1376^ (2C10). In the epitope SS6, substitution ^766^P > L^766^ was identified in IBT/VN/2018, HUA PED45 and HUA PED47 strains isolated in Vietnam only while it was not detected in other strains investigated.

**Figure 3:**
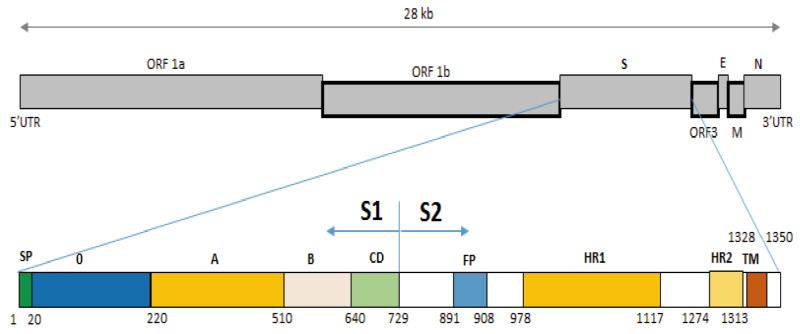
Structure diagram of the spike protein of the PEDV. Diagram depicting the feature domains of the PEDV S protein, including putative cleavage site between S1 and S2 domain at position aa 729. The S1 domain contained the signal peptide subdomain (SP, residues aa 1–19), S0 subdomain (residues aa 20–219), and SA to SD subdomain: SA (residues aa 220–509), SB (residues aa 510–639), SCD (residues aa 640–729). The S2 domain included: the fusion peptide (FP; residues aa 891–908), heptad repeat region 1 (HR1, aa 978–1,117), heptad repeat region 2 (HR2, aa 1,274–1,313), and the transmembrane domain (TM, aa 1,328–1,350).

**Figure 4:**
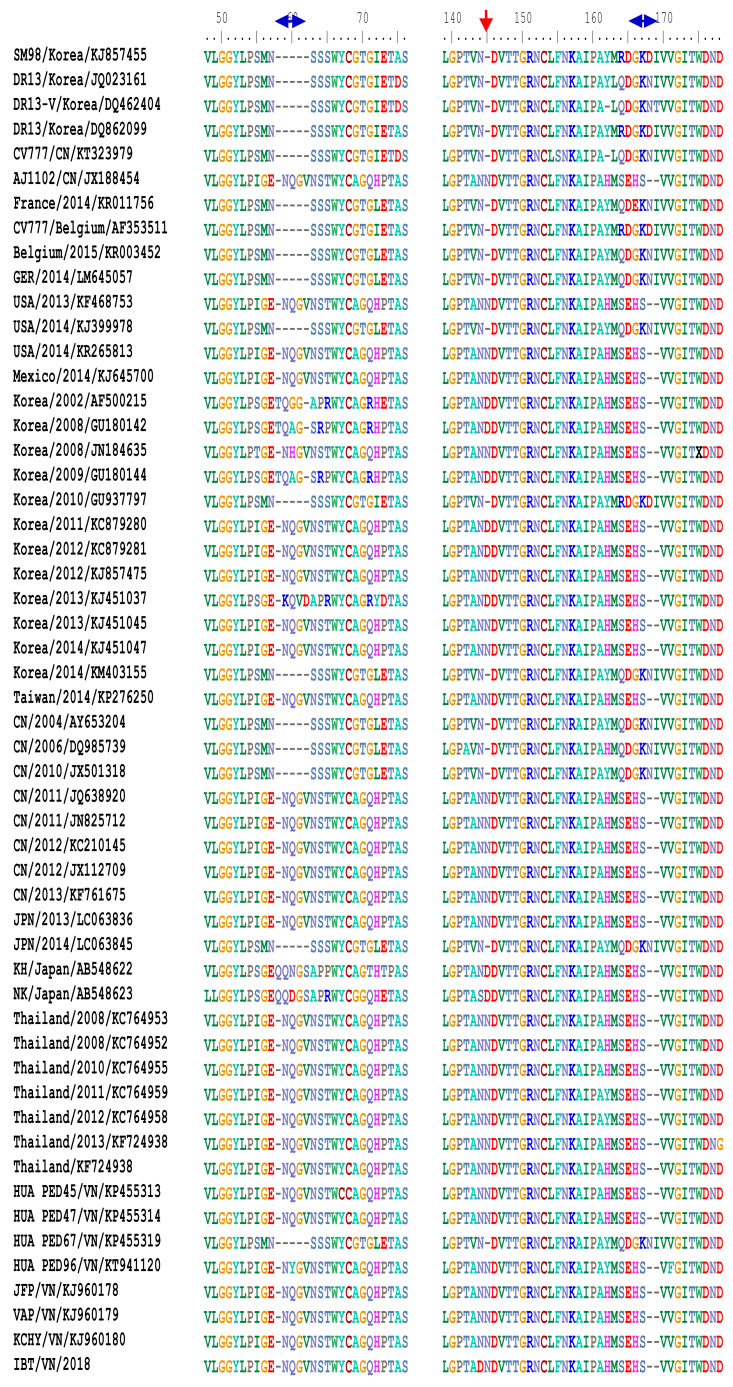
Multiple sequence alignment of the deduced amino acid sequences of the S proteins. The S protein of the IBT/VN/2018 strain and PEDV strains from other countries was aligned and revealed two insertions and one deletion in S protein of IBT/VN/2018 strain at aa positions 59-62, 145, and 168-169 in comparison with the vaccine strains CV777/CN, SM98/Korea and DR13/Korea.

The S protein can also be divided into two domains: S1 (aa 1–789), S2 (aa 790–1,385), or subdomains as described by Li et al. (2016) (Fig. 3). In the S^0^ subdomain of the IBT/VN/2018 strain, two substitutions (^135^DN^136^ > ^135^SI^136^ and ^144^N > D^144^) were found when compared to the vaccine strains (CV777/CN/KT323979, AJ1102/CN/JX188454, SM98/Korea/KJ857455, and DR13/Korea/JQ023162) (Fig. S1). In the S^A^ subdomain, there were eight substitutions between the IBT/VN/2018 strain and the vaccine strains (CV777/CN/KT323979, AJ1102/CN/JX188454, SM98/Korea/KJ857455, and DR13/Korea/JQ023162) at aa positions: ^294^I > M^294^, ^318^A > S^318^, ^335^V > I^335^, ^361^A > T^361^, ^497^R > T^497^, ^501^SH^502^ > ^501^IY^502^, ^506^I > T^506^, ^682^V > I^682^, and ^777^P > L^777^. The IBT/VN/2018 strain also had two insertions (at aa position ^59^NQGV^62^ and ^145^N) and one deletion (^168^DI^169^) in S protein when compared with the CV777/CN/KT323979, AJ1102/CN/JX188454, SM98/Korea/KJ857455, and DR13/Korea/JQ023162 strains (Fig. 4).

The S2 domain presents the typical structural features of class I fusion proteins, including a hydrophobic fusion peptide (FP, residues 891–908), two heptad repeat regions (HR1, residues 978–1,117 and HR2, residues 1,274–1,313), and a C-terminal transmembrane domain (TM, residues 1,328–1,350). In the S2 domain of IBT/VN/2018 strain, eight substitutions at aa positions ^857^V > A^857^, ^1009^L > M^1009^, ^1089^S > L^1089^, ^1207^T > D^1207^, ^1221^F > Y^1221^, ^1229^S > G^1229^, ^1251^D > E^1251^, and ^1279^P > S^1279^ were detected in comparison to the CV777/CN/KT323979, AJ1102/CN/JX188454, SM98/Korea/KJ857455, and DR13/Korea/JQ023162 strains. Among them, two substitutions were found in subdomain S^HR1^ at aa position ^1009^L > M^1009^ and ^1089^S > L^1089^, and one substitution at aa ^1279^P > S^1279^ in subdomain S^HR2^.

The N protein of the IBT/VN/2018 strain had a genetic identity at the nt and the aa sequence with other strains ranging from 92.5%–99.0% and 93.4%–98.8%, respectively (Table S3). The IBT/VN/2018 strain had the highest genetic identity with CH Hubei/CN/2016/KY928065 strain and the lowest identity with the vaccine strain CV777/CN/KT323979. Notably, two substitutions at aa ^364^V > I^364^ and ^378^N > S^378^ were only detected in the IBT/VN/2018 strain (Fig. S2).

The identity of nucleotide sequence and the amino acid sequence of the ORF3 region of the IBT/VN/2018 strain was 89.1%–99.4% and 90.1%–100% compared to other strains, respectively (Table S4). Based on the nucleotide sequence of the ORF3 region, the IBT/VN/2018 strain was most closely related to the CH Hubei/CN/2016/KY928065 strain, sharing a sequence identity of 99.4%. Remarkably, it shared the lowest nucleotide identity (89.1%) with the vaccine strain CV777/CN/KT323979 and JS2008/CN/KC109141 strain. In the ORF3 region, four substitutions were detected at aa positions ^25^L > S^25^, ^70^I > V^70^, ^107^C > F^107^, and ^168^D > N^168^ in IBT/VN/2018, KCHY, VAP, and JFP strains and some other strains such as GD1/CN/2011/JX647847, GDA/CN/2012/JX112709, CH Hubei/CN/2016/KY928065, and SBR1/Thailand/2014/KR610993 (Fig. S3).

## Discussion

In this study, the complete genome of the PEDV strain (IBT/VN/2018) isolated in a severe outbreak in North Vietnam 2018 has been sequenced and analyzed. The genome sequence analysis indicated that the IBT/VN/2018 strain carried specific structural characteristics of members of the *Coronaviridae* family, with genetic identities ranging from 95.7% to 99.3% compared to other PEDV strains (Table S1). The PEDV, based on the S gene sequence, was classified into two genogroups (G1 and G2) and each can sub-divided into groups (G1a, G1b, G2a, and G2b). Classical strains are designated G1a, and the new variant strains (with insertion and deletion in the S gene) belong to G1b. The G2a and G2b include the highly virulent strains in Asia and North America. The phylogenetic tree constructed from the whole genome sequences (Fig. 1) and the S gene sequences (Fig. 2) showed that the IBT/VN/2018 strain belonged to the G2b subgroup including Northern American and Asian S-INDEL strains. According to previous studies, strains that belonged to the G2b and Northern American and Asian S-INDEL subgroup were highly virulent (Lee, 2015; Lin et al., 2016). This result is consistent with previous studies on PEDV strains isolated in Northern provinces of Vietnam which also showed that Vietnamese PEDV strains belong to the G2b subgroup (Kim et al., 2015; Diep et al., 2018).

The S protein composes of 1,383 to 1,386 amino acids consisting of the S1 subunit (aa 1–789) and the S2 subunit (aa 790–1,383) (Fig. 3), depending on the strain (Sun et al., 2007). S protein is known to mediate viral entry and inducing neutralizing antibodies in the natural host. The S1 subunit is the extracellular domain and can bind to target cell receptors (Deng et al., 2016). It is important for cell membrane fusion and virus entry and it is the antigenic target of neutralizing antibodies (Sun et al., 2008; Lee et al., 2011; Deng et al., 2016). It contains a putative signal peptide (aa 1–24), a large extracellular region contains two subdomains: NTD (aa 21–324) and CTD (aa 253–638) (Li, 2015), a single transmembrane domain (aa 1,334–1,356), and a short cytoplasmic tail (Oh et al., 2014). Four neutralizing epitopes (aa 499–638, 748–755, 764–771 designated as COE, SS2, SS6 in domain S1, and 2C10 1,368–1,374 in domain S2) have been determined on the surface of S protein (Sun et al., 2008; Li et al., 2016; Okda et al., 2017). The two regions including SS2 and 2C10 are highly conserved. In contrast, there are two positions at aa 499 and 520 in the COE subdomain and one position at aa 766 in the SS6 subdomain are highly variable (Chang et al., 2002; Cruz, Kim & Shin, 2008; Sun et al., 2008; Lara-Romero et al., 2018). These findings are the basis for the development of new effective vaccines in the future (Yu et al., 2020). Further analysis of sequences in functional protein regions of the IBT/VN/2018 strain showed that at the N-terminal of S protein there were two aa insertions and one aa deletion compared to the CV777/CN/KT323979, AJ1102/CN/JX188454, SM98/Korea/KJ857455, and DR13/Korea/JQ023162 strains (Fig. 4). The substitution at aa ^766^P > L^766^ was also found in the IBT/VN/2018 strain in the epitope SS6. These changes may lead to the ineffectiveness of the drugs and vaccines.

In this study, many substitutions were found on the S protein of the IBT/VN/2018 strain in comparison to the CV777/CN/KT323979, AJ1102/CN/JX188454, SM98/Korea/KJ857455, and DR13/Korea/JQ023162 strains. Among 19 aa substitutions in S protein, the IBT/VN/2018 strain had eleven changes that were similarly in other Vietnamese PEDV strains (KCHY, VAP, JFP and/or HUA PED45, HUA PED47, HUA PED67, and HUA PED96) and some strains from China (CN/2011/JQ638920, CN/2012/JX112709, and CN/2013/KF761675), Korea (Korea/2010/GU937797 and Korea/2011/KC879280), and Thailand (Thailand/2011/KC764959). Remarkably, we found seven substitutions that were only found in the IBT/VN/2018 strain in comparison to other PEDV strains including ^144^N > D^144^, ^318^A > S^318^, ^335^V > I^335^, ^501^SH^502^ > ^501^IY^502^, ^682^V > I^682^, ^1009^L > M^1009^, and ^1089^S > L^1089^ (Fig. S1). The results of phylogenetic analysis showed that the IBT/VN/2018 strain was closely related to PEDV strains in Asia but differ from US and European strains. This can be speculated that the Vietnamese strains have been genetically changes to adapt to environmental conditions and it leads to the reduction of the effectiveness of the vaccine. Our hypothesis was further supported by a recent study which revealed that the mutations in the neutralizing epitope regions in the S gene cause inefficiencies in vaccination (Li et al., 2013). These regions were detected at aa 7–146 and 271–278 in the neutralizing epitopes (Li et al., 2013). However, more studies need to be carried out to confirm this assumption.

S^0^ also known as the N-terminal region is a functional receptor for the porcine epidemic diarrhea virus. PEDV uses the N-terminal region as the major receptor for cell entry (Li et al., 2017). The N-terminal region binds to sugar which acts as its co-receptor. This process is the first important step to help the virus penetrate cells (Deng et al., 2016). Recent reports suggest that any amino acid mutation can change the virulence of Coronavirus (Peng et al., 2011; Wu et al., 2012; Teenavechyan et al., 2016; Qin et al., 2019; Wabalo et al., 2021). Consequently, the N-terminal domain can be used as a vaccination strategy to prevent PEDV infections. The S^0^ subdomain of the IBT/VN/2018 strain had two aa substitutions at positions ^135^DN^136^ > ^135^SI^136^ and N^144^ > D^144^ which may make virus entry into cells easier. In other words, these mutations can enhance the pathogenicity of the Vietnamese strain. However, this needs to be experimentally confirmed.

In the IBT/VN/2018 strain, we also identified two aa substitutions in the S^HR1^ subdomain at aa positions ^1009^L > M^1009^ and ^1089^S > L^1089^, and one substitution at aa ^1279^P > S^1279^ in subdomain S^HR2^. In Coronavirus, membrane fusion is initiated by the invention and intervention of the fusion protein (FP) into the target cell membrane, then the fusion protein combined with HR1 and HR2 regions form a stable structure. This process took the virus’s transmembrane domain accessed and fused it with the membrane of the host cell (Eckert & Kim, 2001; Li et al., 2016). Therefore, the substitutions in the functional areas may lead to change this process and interferes with the viral entry.

Recent reports showed that the S protein, the N protein and the accessory ORF3 protein also play an important role in regulating the virulence of PEDV strains and can cause severe damage by evading host immune mechanisms (Zhang & Yoo, 2016; Kim et al., 2020; Li et al., 2020). The N protein is identified as a multifunctional region and participating in many stages in viral replication and regulating functions of PEDV (Zuniga et al., 2010; Liwnaree et al., 2019). Two novel epitopes at aa 18–133 and 252–262 were identified on the N protein (Wang et al., 2016a). The accessory protein ORF3 was identified as an ion channel and has many regulatory functions (Wang et al., 2012; Wang et al., 2016b; Kaewborisuth, He & Jongkaewwattana, 2018). Kaewborisuth, He & Jongkaewwattana (2018) indicated that the ORF3 protein can work together with the S protein for PEDV assembly at the viral replication step. The ORF3 protein is also related to the virulence of PEDV (Song et al., 2003; Park et al., 2007; Chen et al., 2013b) through its regulatory process in viral production (Wang et al., 2012). In general, the ORF3 protein is an important virulence gene for PEDV pathogenicity, and molecular epidemiology studies of PEDV (Pospischil, Stuedli & Kiupel, 2002; Song et al., 2003; Shirato et al., 2011). Two substitutions at aa positions ^364^N > I^364^ and ^378^N > S^378^ and four substitutions at aa positions ^25^L > S^25^, ^70^I > V^70^, ^107^C > F^107^, and ^168^D > N^168^ were found in the N and ORF3 region of PEDV-VN strains (IBT/VN/2018, KCHY, VAP, and JFP) (Figs. S2, S3) suggesting that these PEDV-VN strains have evoluted and changed their pathogenicity.

The results of homologous modeling showed that the acquired mutations found in S protein of the IBT/VN/2018 strain including mutations in the S^0^ domain does not remarkably affect its overall 3D structure (Fig. S4). This data is well consistent with result of a previous study which indicated that the deletion of S^0^ domain does not impart any macroscopic changes in spike protein conformation (Kirchdoerfer et al., 2021).

It has been known that glycosylation plays an important role receptor binding, virus entry, protein proteolysis, and protein transport, thereby altering the virulence and immune evasion of virus (Vigerust & Shepherd, 2007). The glycosylation sites have been reported in PEDV in recent years (Kim et al., 2015; Wen et al., 2021). Six glycosylation sites (25^G+^, 123^N+^, 62^V +^, 144^D+^, 1009^M+^, and 1279^L+^) were detected in the ORF3, N, and S proteins of the IBT/VN/2018 strain when compared with the vaccine strains. Among that three novel glycosylation sites (144^D+^, 1009^M+^, and 1279^L+^) were detected in the IBT/VN/2018 strain. As a consequence, the amino acid substitutions in the S, N and ORF3 proteins may help the IBT/VN/2018 strain evade the host immune system and probably change the virulence of the virus.

## Conclusion

Our study showed that the IBT/VN/2018 strain belonged to the G2b subgroup that along with the Northern American and Asian S-INDEL strains and are considered as a highly virulent group. Remarkably, eight amino acid substitutions (^294^I > M^294^, ^318^A > S^318^, ^335^V > I^335^, ^361^A > T^361^, ^497^R > T^497^, ^501^SH^502^ > ^501^IY^502^, ^506^I > T^506^, ^682^V > I^682^, and ^777^P > L^777^) were found in S^A^ subdomain in the Vietnamese strain compared with the vaccine strains. In addition to these substitutions, three novel N- and O-glycosylation sites (144^D+^, 1009^M+^, and 1279^L+^) were detected in the S protein of IBT/VN/2018 strain. The continual mutations in these genes may have generated a novel antigenic strain and help the virus escape from the host immune response induced by the vaccine. Our results highlight the importance of molecular characterization of PEDV strains circulating in Vietnam and provide a molecular potential for the development of an effective vaccine to control PEDV infections of pigs in Vietnam.

## Supplemental Information

10.7717/peerj.12329/supp-1 Supplemental Information 1 Comparison of amino acid sequences of S gene The blue arrow pointed the insertions (at aa positions ^59^NQGV^62^ and ^145^N), deletion (at aa position ^168^DI^169^), and the substitutions at aa ^135^DN^136^ > ^135^SI^136^, ^497^R > T^497^, ^506^I > T^506^, ^857^V > A^857^, ^1221^F > Y^1221^, and ^1279^P > S^1279^ in S protein of IBT/VN/2018 strain when comparison with the vaccine strains CV777/CN, SM98/Korea and DR13/Korea. The red arrow pointed the substitutions at aa ^144^N > D^144^, ^294^I > M^294^, ^318^A > S^318^, ^335^V > I^335^, ^361^A > T^361^, ^501^SH^502^ > ^501^IY^502^, ^682^L > F^682^, ^777^P >  L^77^^7^, ^1009^L > M^1009^, ^1089^S > L^1089^, ^1207^T > D^1207^, ^1229^S > G^1229^, and ^1251^D > E^12^^51^ in S protein of IBT/VN/2018 strain when comparison with the vaccine strains AJ1102/CN, CV777/CN, SM98/Korea and DR13/Korea.

10.7717/peerj.12329/supp-2 Supplemental Information 2 Comparison of amino acid sequences of N gene The blue arrow pointed the changes at aa positions ^216^V > M^216^, ^400^E > D^400^ in PEDV-VN strains (IBT/VN/2018, KCHY, VAP, and JFP) and some of PEDV strains (from China and Thailand) compared to the vaccine strains CV777/CN and DR13/Korea. The green arrow pointed the changes at aa positions ^364^V > I^364^ and ^378^N > S^378^ between IBT/VN/2018 strain and other strains.

10.7717/peerj.12329/supp-3 Supplemental Information 3 Comparison of amino acid sequences of ORF3 gene There were four substitutions at aa positions ^25^L > S^25^, ^70^I > V^70^, ^107^C > F^107^, and ^168^D > N^168^ that found in PEDV-VN (IBT/VN/2018, KCHY, VAP, and JFP) strains and GD1/CN/2011/JX647847, GDA/CN/2012/JX112709, CH Hubei/CN/2016/KY928065, and CBR1/Thailand/2014/KR610993 strains compared to other strains.

10.7717/peerj.12329/supp-4 Supplemental Information 4 Homology modeling predicts the 3D structure of S protein of IBT/VN/2018 through sequence alignment of the known 3D structure strain (6U7K) No changes were observed in the 3D structure of the S protein of IBT/VN/2018 at mutated sites p.P766L, p.L1009M, and p.S1089L as compared to 6U7K strain.

10.7717/peerj.12329/supp-5 Supplemental Information 5 Genetic similarity of complete nucleotide sequences (%) between the IBT/VN/2018 and other reference sequences

10.7717/peerj.12329/supp-6 Supplemental Information 6 Genetic similarity of nucleotide sequences and amino acid sequences for the coding region of the Spike protein (%) between the IBT/VN/2018 and other reference sequences

10.7717/peerj.12329/supp-7 Supplemental Information 7 Genetic similarity of nucleotide sequences and amino acid sequences for the coding region of the Nucleocapsid protein (%) between the IBT/VN/2018 and other reference sequences

10.7717/peerj.12329/supp-8 Supplemental Information 8 Genetic similarity of nucleotide sequences and amino acid sequences for the coding region of the ORF3 (%) between the IBT/VN/2018 and other reference sequences

## Additional Information and Declarations

### Field Study Permissions

The following information was supplied relating to field study approvals (i.e., approving body and any reference numbers): The Institute of Biotechnology, Vietnam Academy of Science and Technology approved the collection of postmortem veterinary samples from farms.

### DNA Deposition

The following information was supplied regarding the deposition of DNA sequences: The data are available at NCBI: MT198679.

### Data Availability

The following information was supplied regarding data availability: The raw measurements are available in the Supplemental Files.
