# Supplementary material for: Changes in the spike and nucleocapsid protein of porcine epidemic diarrhea virus strain in Vietnam—a molecular potential for the vaccine development?
Source: PeerJ. 2021 Oct 18;9:e12329. doi: 10.7717/peerj.12329 (PMC8530102; doi:10.7717/peerj.12329)
Supplement: Supplemental Information 1 — The blue arrow pointed the insertions (at aa positions 59NQGV62 and 145N), deletion (at aa position 168DI169), and the substitutions at aa 135DN136 > 135SI136, 497R > T497, 506I > T506, 857V > A857, 1221F > Y1221, and 1279P > S1279 in S protein of IBT/VN/2018 strain when comparison with the vaccine strains CV777/CN, SM98/Korea and DR13/Korea. The red arrow pointed the substitutions at aa 144N > D144, 294I > M294, 318A > S318, 335V > I335, 361A > T361, 501SH502 > 501IY502, 682L > F682, 777P > L777, 1009L > M1009, 1089S > L1089, 1207T > D1207, 1229S > G1229, and 1251D > E1251 in S protein of IBT/VN/2018 strain when comparison with the vaccine strains AJ1102/CN, CV777/CN, SM98/Korea and DR13/Korea. [file peerj-09-12329-s001.pdf]

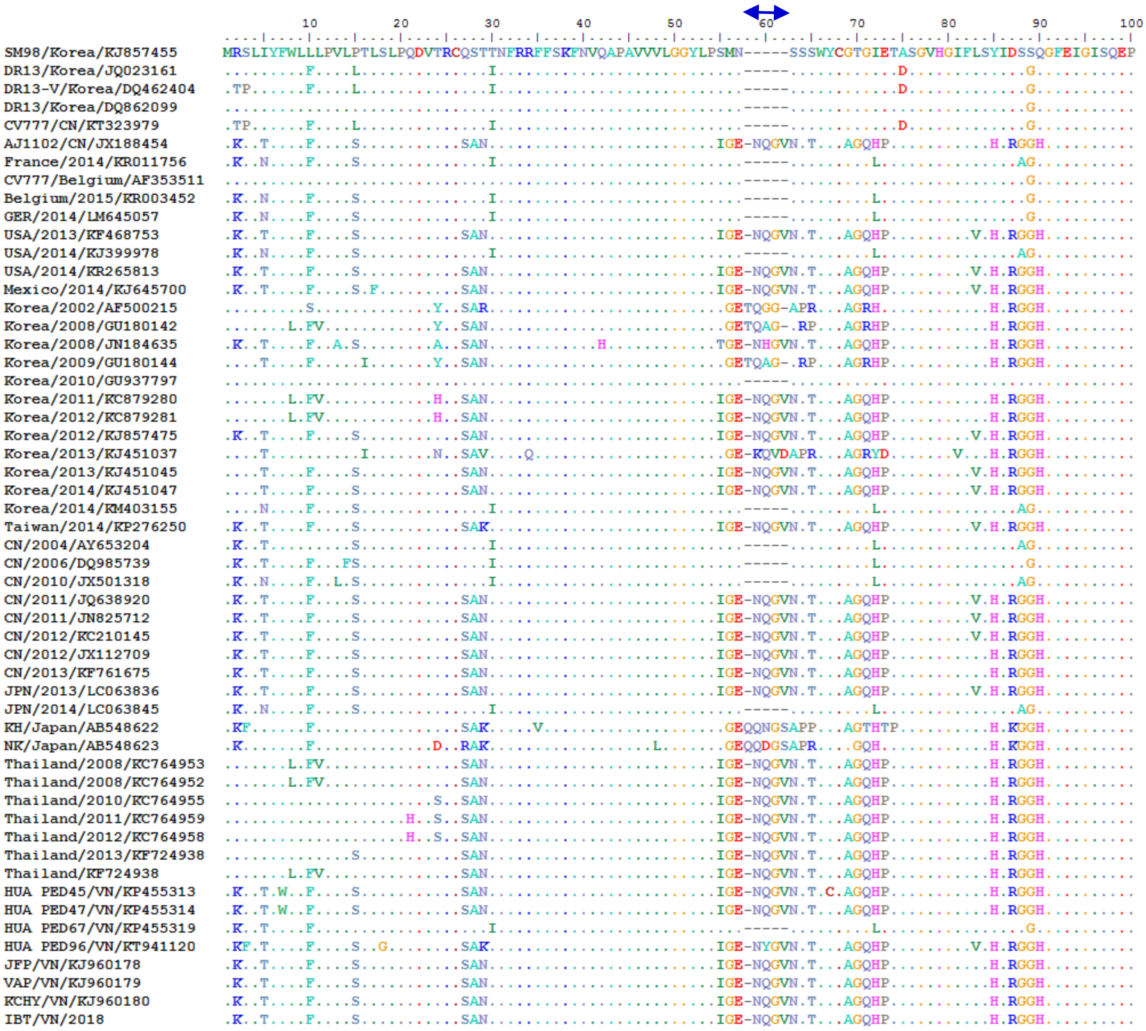
10
20
30
40
50
60
70
80
90
100
SM98/Korea/KJ857455
MRSLIYFWLLLPVLPTLSLPQDVTRCQSTTNFRRFFSKFNVQAPAVVVLGGYLPSMN-----SSSWYCGTGIETASGVHGIFLSYIDSSQGFEIGISQEP
DR13/Korea/JQ023161
DR13-V/Korea/DQ462404
DR13/Korea/DQ862099
CV777/CN/KT323979
AJ1102/CN/JX188454
France/2014/KR011756
CV777/Belgium/AF353511
Belgium/2015/KR003452
GER/2014/LM645057
USA/2013/KF468753
USA/2014/KJ399978
USA/2014/KR265813
Mexico/2014/KJ645700
Korea/2002/AF500215
Korea/2008/GU180142
Korea/2008/JN184635
Korea/2009/GU180144
Korea/2010/GU937797
Korea/2011/KC879280
Korea/2012/KC879281
Korea/2012/KJ857475
Korea/2013/KJ451037
Korea/2013/KJ451045
Korea/2014/KJ451047
Korea/2014/KM403155
Taiwan/2014/KP276250
CN/2004/AY653204
CN/2006/DQ985739
CN/2010/JX501318
CN/2011/JQ638920
CN/2011/JN825712
CN/2012/KC210145
CN/2012/JX112709
CN/2013/KF761675
JPN/2013/LC063836
JPN/2014/LC063845
KH/Japan/AB548622
NK/Japan/AB548623
Thailand/2008/KC764953
Thailand/2008/KC764952
Thailand/2010/KC764955
Thailand/2011/KC764959
Thailand/2012/KC764958
Thailand/2013/KF724938
Thailand/KF724938
HUA PED45/VN/KP455313
HUA PED47/VN/KP455314
HUA PED67/VN/KP455319
HUA PED96/VN/KT941120
JFP/VN/KJ960178
VAP/VN/KJ960179
KCHY/VN/KJ960180
IBT/VN/2018

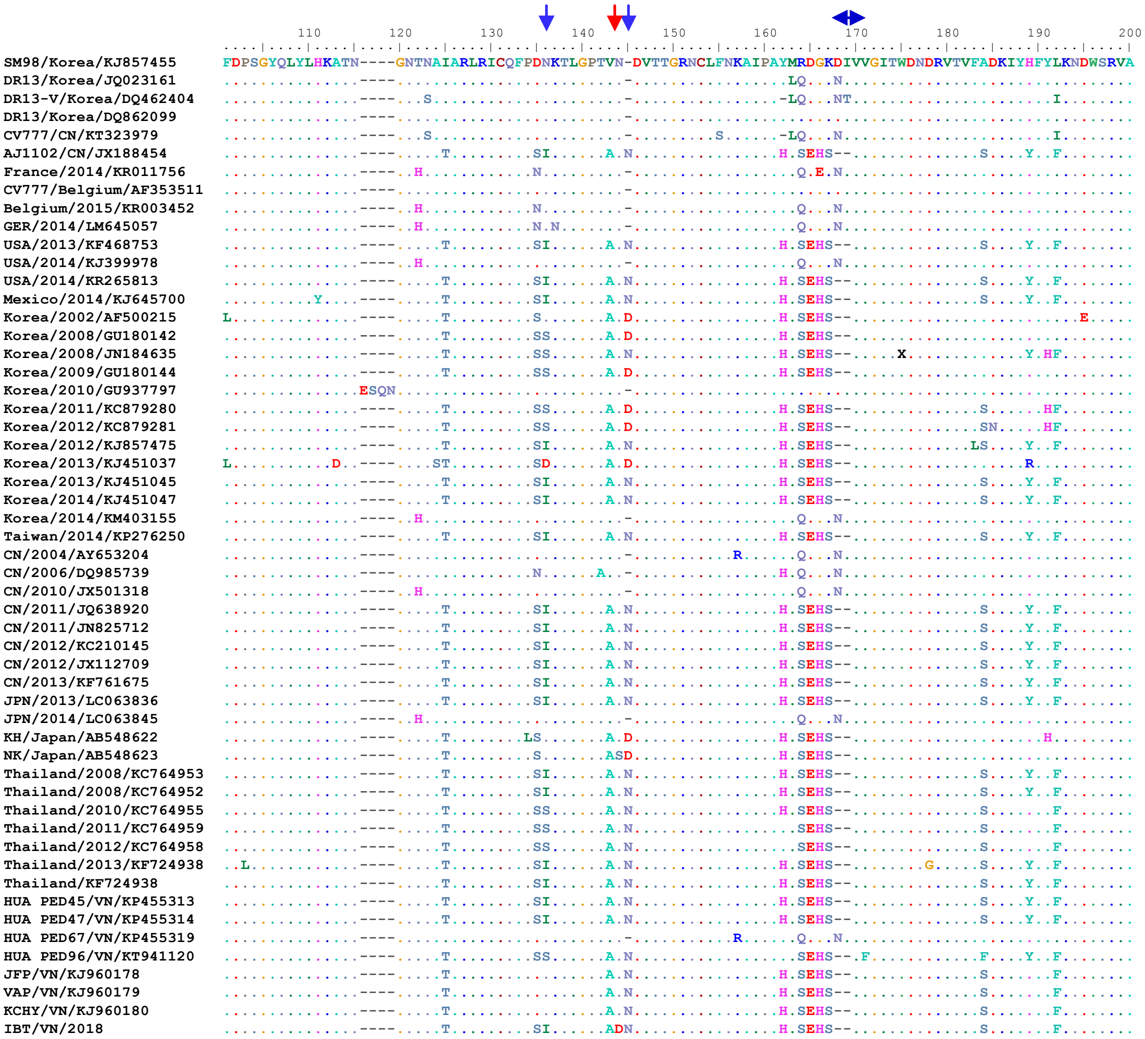
110 120 130 140 150 160 170 180 190 200
SM98/Korea/KJ857455 FDPSGYQLYLHKATN----GNTNAIARLRICQFPDNKTLGPTVN-DVTTGRNCLFNKAIPAYMRDGKDIVVGITWDNDRVTVFADKIYHFYLKNDWSRVA
DR13/Korea/JQ023161
DR13-V/Korea/DQ462404
DR13/Korea/DQ862099
CV777/CN/KT323979
AJ1102/CN/JX188454
France/2014/KR011756
CV777/Belgium/AF353511
Belgium/2015/KR003452
GER/2014/LM645057
USA/2013/KF468753
USA/2014/KJ399978
USA/2014/KR265813
Mexico/2014/KJ645700
Korea/2002/AF500215
Korea/2008/GU180142
Korea/2008/JN184635
Korea/2009/GU180144
Korea/2010/GU937797
Korea/2011/KC879280
Korea/2012/KC879281
Korea/2012/KJ857475
Korea/2013/KJ451037
Korea/2013/KJ451045
Korea/2014/KJ451047
Korea/2014/KM403155
Taiwan/2014/KP276250
CN/2004/AY653204
CN/2006/DQ985739
CN/2010/JX501318
CN/2011/JQ638920
CN/2011/JN825712
CN/2012/KC210145
CN/2012/JX112709
CN/2013/KF761675
JPN/2013/LC063836
JPN/2014/LC063845
KH/Japan/AB548622
NK/Japan/AB548623
Thailand/2008/KC764953
Thailand/2008/KC764952
Thailand/2010/KC764955
Thailand/2011/KC764959
Thailand/2012/KC764958
Thailand/2013/KF724938
Thailand/KF724938
HUA PED45/VN/KP455313
HUA PED47/VN/KP455314
HUA PED67/VN/KP455319
HUA PED96/VN/KT941120
JFP/VN/KJ960178
VAP/VN/KJ960179
KCHY/VN/KJ960180
IBT/VN/2018

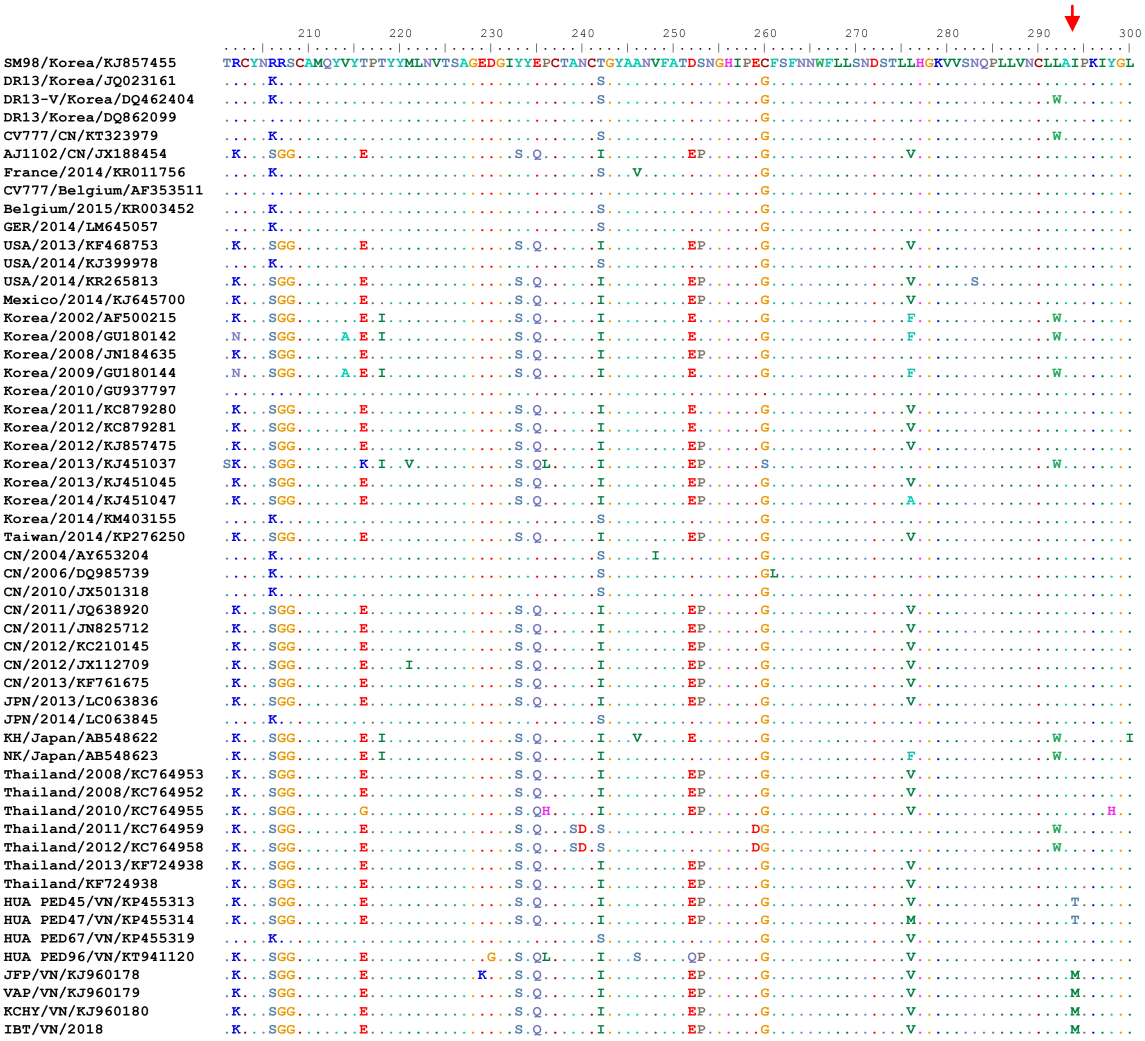

210
220
230
240
250
260
270
280
290
300
SM98/Korea/KJ857455 TRCYNRRSCAMQYVYTPTYYMLNVTSAGEDGIYYEPCTANCTGYAANVFATDSNGHIPECFSFNNWFLLSNDSTLLHGKVVSNQPLLVNCLLAIPKIYGL
DR13/Korea/JQ023161
DR13-V/Korea/DQ462404
DR13/Korea/DQ862099
CV777/CN/KT323979
AJ1102/CN/JX188454
France/2014/KR011756
CV777/Belgium/AF353511
Belgium/2015/KR003452
GER/2014/LM645057
USA/2013/KF468753
USA/2014/KJ399978
USA/2014/KR265813
Mexico/2014/KJ645700
Korea/2002/AF500215
Korea/2008/GU180142
Korea/2008/JN184635
Korea/2009/GU180144
Korea/2010/GU937797
Korea/2011/KC879280
Korea/2012/KC879281
Korea/2012/KJ857475
Korea/2013/KJ451037
Korea/2013/KJ451045
Korea/2014/KJ451047
Korea/2014/KM403155
Taiwan/2014/KP276250
CN/2004/AY653204
CN/2006/DQ985739
CN/2010/JX501318
CN/2011/JQ638920
CN/2011/JN825712
CN/2012/KC210145
CN/2012/JX112709
CN/2013/KF761675
JPN/2013/LC063836
JPN/2014/LC063845
KH/Japan/AB548622
NK/Japan/AB548623
Thailand/2008/KC764953
Thailand/2008/KC764952
Thailand/2010/KC764955
Thailand/2011/KC764959
Thailand/2012/KC764958
Thailand/2013/KF724938
Thailand/KF724938
HUA PED45/VN/KP455313
HUA PED47/VN/KP455314
HUA PED67/VN/KP455319
HUA PED96/VN/KT941120
JFP/VN/KJ960178
VAP/VN/KJ960179
KCHY/VN/KJ960180
IBT/VN/2018

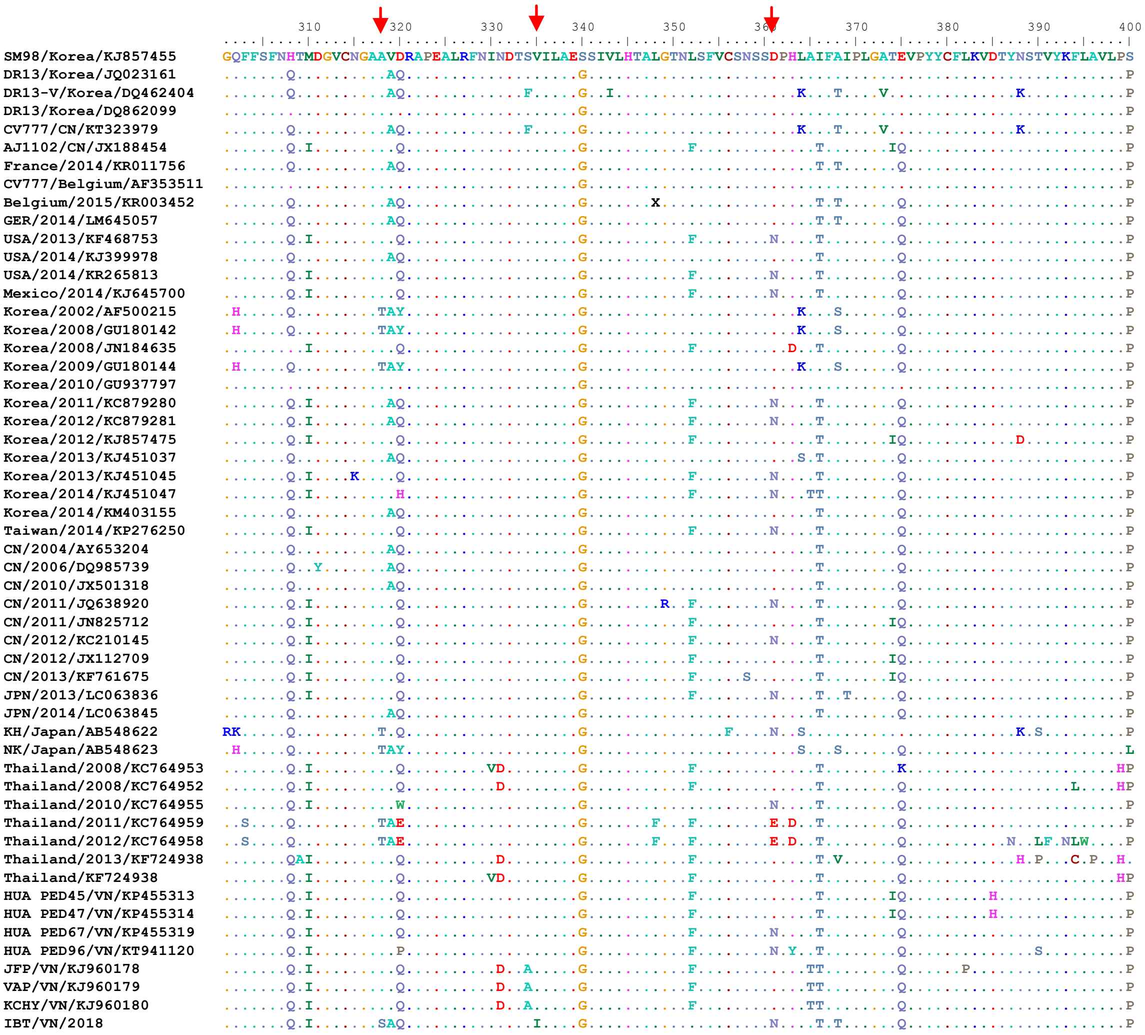
310 320 330 340 350 360 370 380 390 400
SM98/Korea/KJ857455 GQFFSFNHTMDGVCNGAAVDRAPEALRFNINDTSVILAESSIVLHTALGTNLSFVCSNSSDPHLAIFAIPLGATEVPYYCFLKVDTYNSTVYKFLAVLPS
DR13/Korea/JQ023161
DR13-V/Korea/DQ462404
DR13/Korea/DQ862099
CV777/CN/KT323979
AJ1102/CN/JX188454
France/2014/KR011756
CV777/Belgium/AF353511
Belgium/2015/KR003452
GER/2014/LM645057
USA/2013/KF468753
USA/2014/KJ399978
USA/2014/KR265813
Mexico/2014/KJ645700
Korea/2002/AF500215
Korea/2008/GU180142
Korea/2008/JN184635
Korea/2009/GU180144
Korea/2010/GU937797
Korea/2011/KC879280
Korea/2012/KC879281
Korea/2012/KJ857475
Korea/2013/KJ451037
Korea/2013/KJ451045
Korea/2014/KJ451047
Korea/2014/KM403155
Taiwan/2014/KP276250
CN/2004/AY653204
CN/2006/DQ985739
CN/2010/JX501318
CN/2011/JQ638920
CN/2011/JN825712
CN/2012/KC210145
CN/2012/JX112709
CN/2013/KF761675
JPN/2013/LC063836
JPN/2014/LC063845
KH/Japan/AB548622
NK/Japan/AB548623
Thailand/2008/KC764953
Thailand/2008/KC764952
Thailand/2010/KC764955
Thailand/2011/KC764959
Thailand/2012/KC764958
Thailand/2013/KF724938
Thailand/KF724938
HUA PED45/VN/KP455313
HUA PED47/VN/KP455314
HUA PED67/VN/KP455319
HUA PED96/VN/KT941120
JFP/VN/KJ960178
VAP/VN/KJ960179
KCHY/VN/KJ960180
IBT/VN/2018

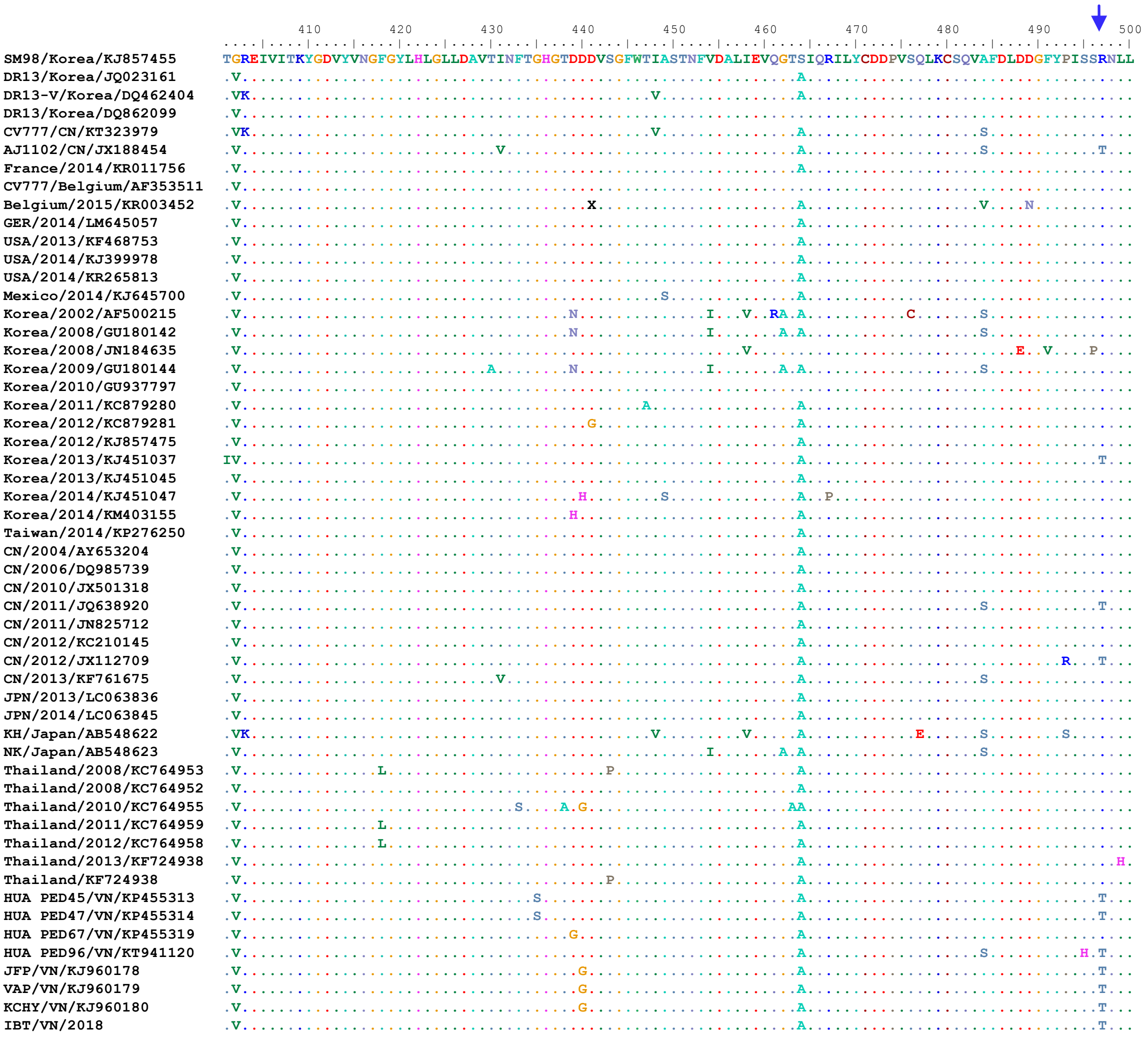
410
420
430
440
450
460
470
480
490
500
SM98/Korea/KJ857455
DR13/Korea/JQ023161
DR13-V/Korea/DQ462404
DR13/Korea/DQ862099
CV777/CN/KT323979
AJ1102/CN/JX188454
France/2014/KR011756
CV777/Belgium/AF353511
Belgium/2015/KR003452
GER/2014/LM645057
USA/2013/KF468753
USA/2014/KJ399978
USA/2014/KR265813
Mexico/2014/KJ645700
Korea/2002/AF500215
Korea/2008/GU180142
Korea/2008/JN184635
Korea/2009/GU180144
Korea/2010/GU937797
Korea/2011/KC879280
Korea/2012/KC879281
Korea/2012/KJ857475
Korea/2013/KJ451037
Korea/2013/KJ451045
Korea/2014/KJ451047
Korea/2014/KM403155
Taiwan/2014/KP276250
CN/2004/AY653204
CN/2006/DQ985739
CN/2010/JX501318
CN/2011/JQ638920
CN/2011/JN825712
CN/2012/KC210145
CN/2012/JX112709
CN/2013/KF761675
JPN/2013/LC063836
JPN/2014/LC063845
KH/Japan/AB548622
NK/Japan/AB548623
Thailand/2008/KC764953
Thailand/2008/KC764952
Thailand/2010/KC764955
Thailand/2011/KC764959
Thailand/2012/KC764958
Thailand/2013/KF724938
Thailand/KF724938
HUA PED45/VN/KP455313
HUA PED47/VN/KP455314
HUA PED67/VN/KP455319
HUA PED96/VN/KT941120
JFP/VN/KJ960178
VAP/VN/KJ960179
KCHY/VN/KJ960180
IBT/VN/2018

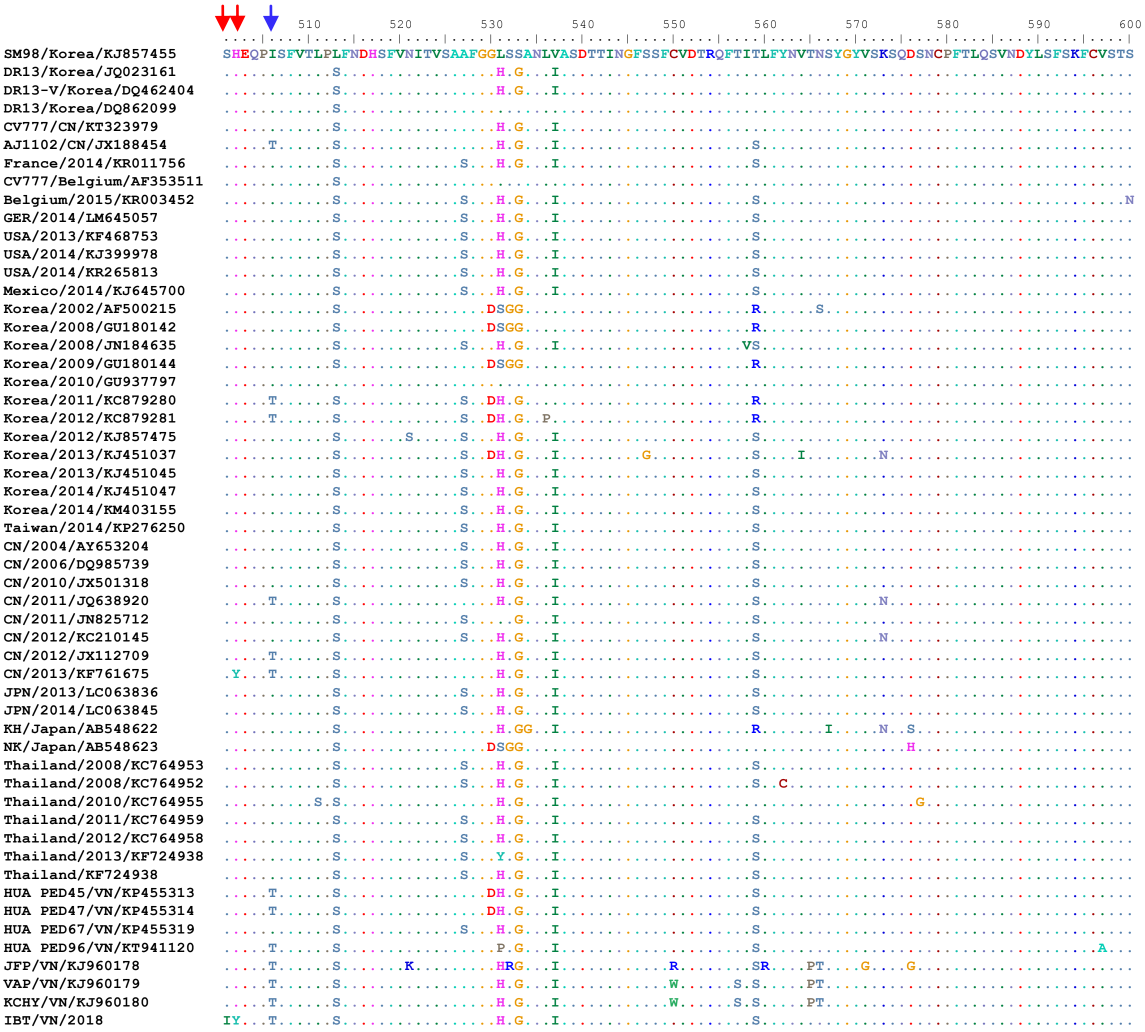

510 520 530 540 550 560 570 580 590 600
SM98/Korea/KJ857455
SHEQPISFVTLPLFNDHSFVNITVSAAFGGLSSANLVASDTTINGFSSFCVDTRQFTITLFYNVTNSYGYVSKSQDSNCPFTLQSVNDYLSFSKFCVSTS
DR13/Korea/JQ023161
DR13-V/Korea/DQ462404
DR13/Korea/DQ862099
CV777/CN/KT323979
AJ1102/CN/JX188454
France/2014/KR011756
CV777/Belgium/AF353511
Belgium/2015/KR003452
GER/2014/LM645057
USA/2013/KF468753
USA/2014/KJ399978
USA/2014/KR265813
Mexico/2014/KJ645700
Korea/2002/AF500215
Korea/2008/GU180142
Korea/2008/JN184635
Korea/2009/GU180144
Korea/2010/GU937797
Korea/2011/KC879280
Korea/2012/KC879281
Korea/2012/KJ857475
Korea/2013/KJ451037
Korea/2013/KJ451045
Korea/2014/KJ451047
Korea/2014/KM403155
Taiwan/2014/KP276250
CN/2004/AY653204
CN/2006/DQ985739
CN/2010/JX501318
CN/2011/JQ638920
CN/2011/JN825712
CN/2012/KC210145
CN/2012/JX112709
CN/2013/KF761675
JPN/2013/LC063836
JPN/2014/LC063845
KH/Japan/AB548622
NK/Japan/AB548623
Thailand/2008/KC764953
Thailand/2008/KC764952
Thailand/2010/KC764955
Thailand/2011/KC764959
Thailand/2012/KC764958
Thailand/2013/KF724938
Thailand/KF724938
HUA PED45/VN/KP455313
HUA PED47/VN/KP455314
HUA PED67/VN/KP455319
HUA PED96/VN/KT941120
JFP/VN/KJ960178
VAP/VN/KJ960179
KCHY/VN/KJ960180
IBT/VN/2018

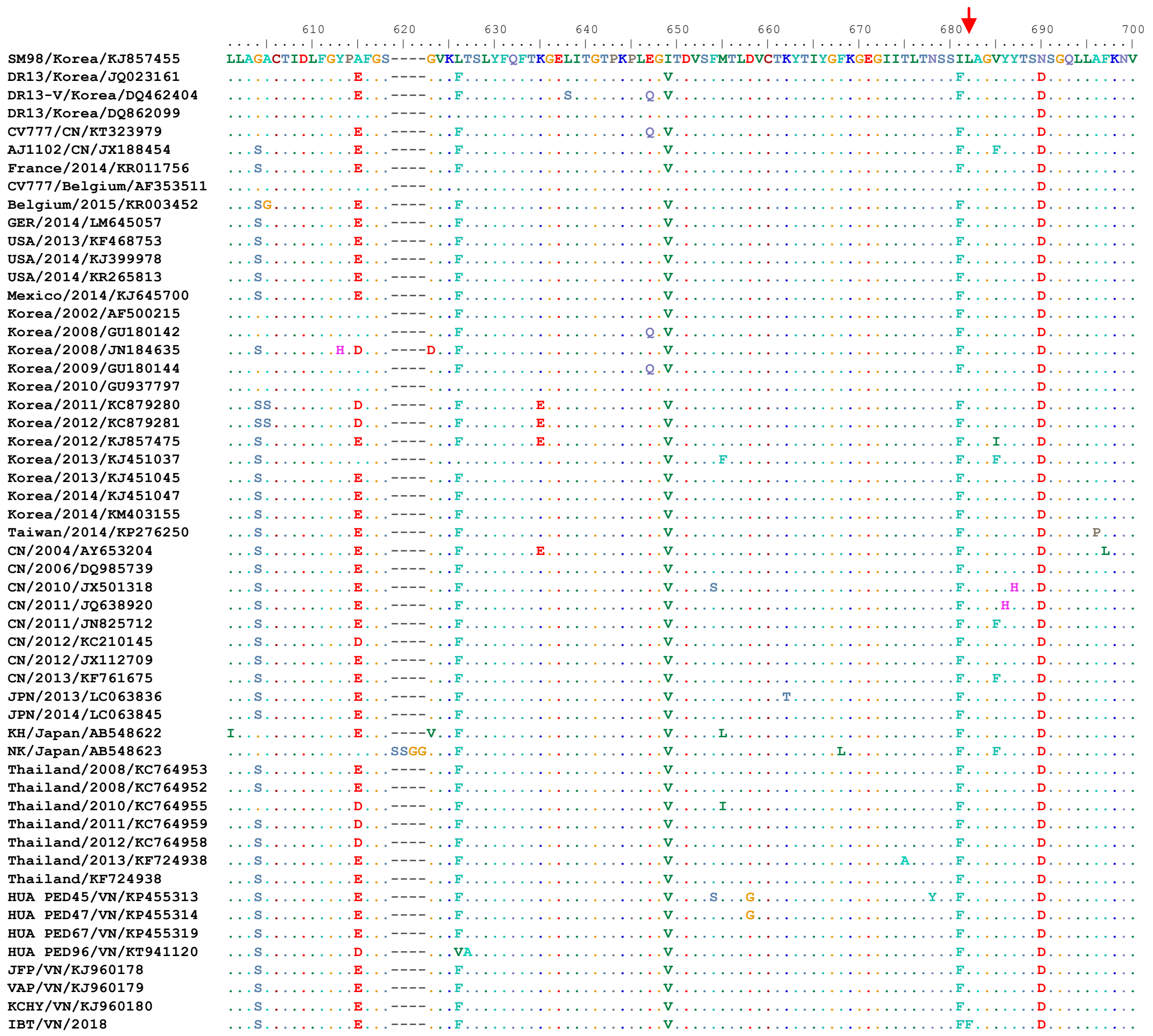
610
620
630
640
650
660
670
680
690
700
SM98/Korea/KJ857455
DR13/Korea/JQ023161
DR13-V/Korea/DQ462404
DR13/Korea/DQ862099
CV777/CN/KT323979
AJ1102/CN/JX188454
France/2014/KR011756
CV777/Belgium/AF353511
Belgium/2015/KR003452
GER/2014/LM645057
USA/2013/KF468753
USA/2014/KJ399978
USA/2014/KR265813
Mexico/2014/KJ645700
Korea/2002/AF500215
Korea/2008/GU180142
Korea/2008/JN184635
Korea/2009/GU180144
Korea/2010/GU937797
Korea/2011/KC879280
Korea/2012/KC879281
Korea/2012/KJ857475
Korea/2013/KJ451037
Korea/2013/KJ451045
Korea/2014/KJ451047
Korea/2014/KM403155
Taiwan/2014/KP276250
CN/2004/AY653204
CN/2006/DQ985739
CN/2010/JX501318
CN/2011/JQ638920
CN/2011/JN825712
CN/2012/KC210145
CN/2012/JX112709
CN/2013/KF761675
JPN/2013/LC063836
JPN/2014/LC063845
KH/Japan/AB548622
NK/Japan/AB548623
Thailand/2008/KC764953
Thailand/2008/KC764952
Thailand/2010/KC764955
Thailand/2011/KC764959
Thailand/2012/KC764958
Thailand/2013/KF724938
Thailand/KF724938
HUA PED45/VN/KP455313
HUA PED47/VN/KP455314
HUA PED67/VN/KP455319
HUA PED96/VN/KT941120
JFP/VN/KJ960178
VAP/VN/KJ960179
KCHY/VN/KJ960180
IBT/VN/2018

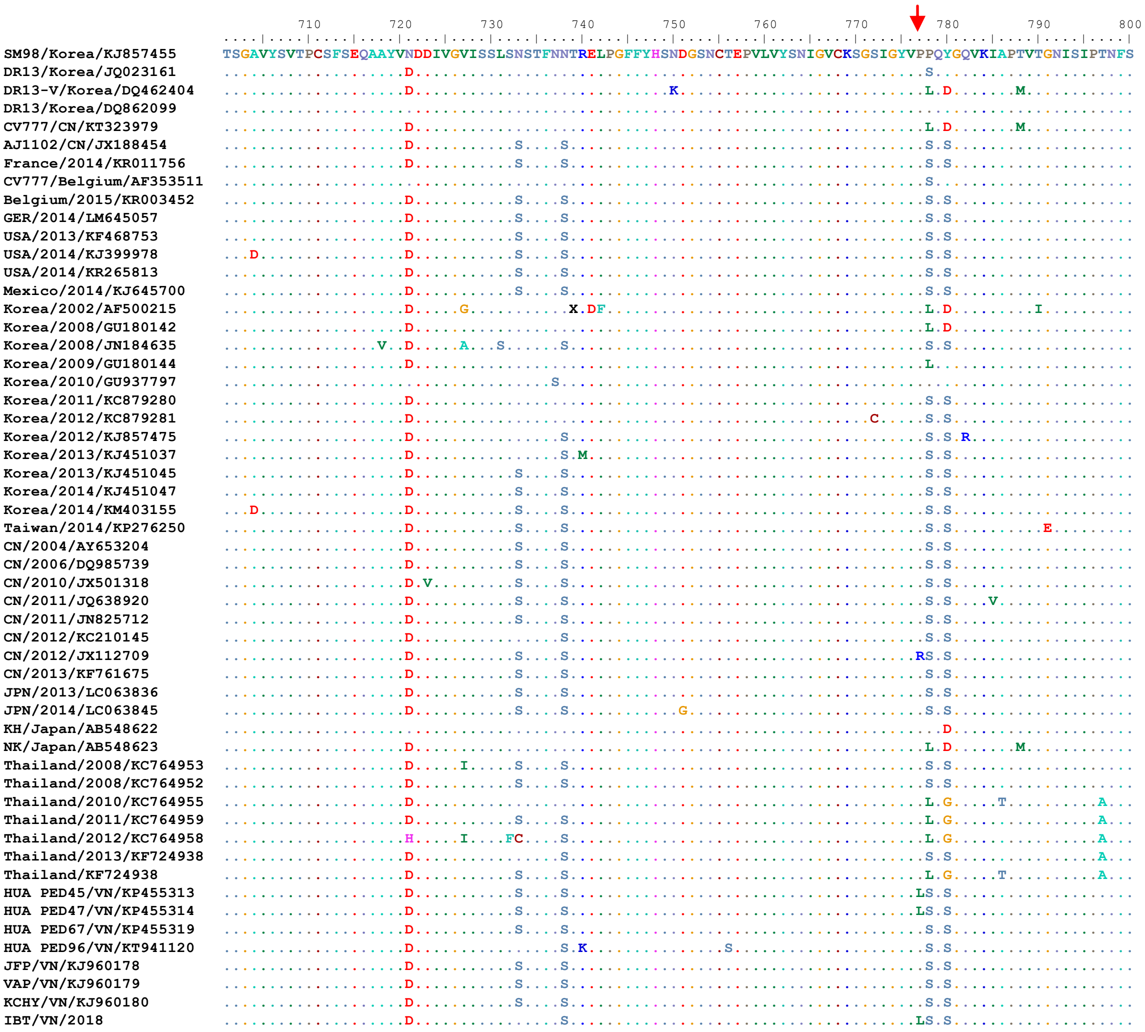
710 720 730 740 750 760 770 780 790 800
SM98/Korea/KJ857455 TSGAVYSVTPCSFSEQAAYVNDDIVGVISSLSNSTFNNTRELPGFFYHSNDGSNCTEPVLVYSNIGVCKSGSIGYVPPQYGQVKIAPTVTGNISIPTNFS
DR13/Korea/JQ023161
DR13-V/Korea/DQ462404
DR13/Korea/DQ862099
CV777/CN/KT323979
AJ1102/CN/JX188454
France/2014/KR011756
CV777/Belgium/AF353511
Belgium/2015/KR003452
GER/2014/LM645057
USA/2013/KF468753
USA/2014/KJ399978
USA/2014/KR265813
Mexico/2014/KJ645700
Korea/2002/AF500215
Korea/2008/GU180142
Korea/2008/JN184635
Korea/2009/GU180144
Korea/2010/GU937797
Korea/2011/KC879280
Korea/2012/KC879281
Korea/2012/KJ857475
Korea/2013/KJ451037
Korea/2013/KJ451045
Korea/2014/KJ451047
Korea/2014/KM403155
Taiwan/2014/KP276250
CN/2004/AY653204
CN/2006/DQ985739
CN/2010/JX501318
CN/2011/JQ638920
CN/2011/JN825712
CN/2012/KC210145
CN/2012/JX112709
CN/2013/KF761675
JPN/2013/LC063836
JPN/2014/LC063845
KH/Japan/AB548622
NK/Japan/AB548623
Thailand/2008/KC764953
Thailand/2008/KC764952
Thailand/2010/KC764955
Thailand/2011/KC764959
Thailand/2012/KC764958
Thailand/2013/KF724938
Thailand/KF724938
HUA PED45/VN/KP455313
HUA PED47/VN/KP455314
HUA PED67/VN/KP455319
HUA PED96/VN/KT941120
JFP/VN/KJ960178
VAP/VN/KJ960179
KCHY/VN/KJ960180
IBT/VN/2018

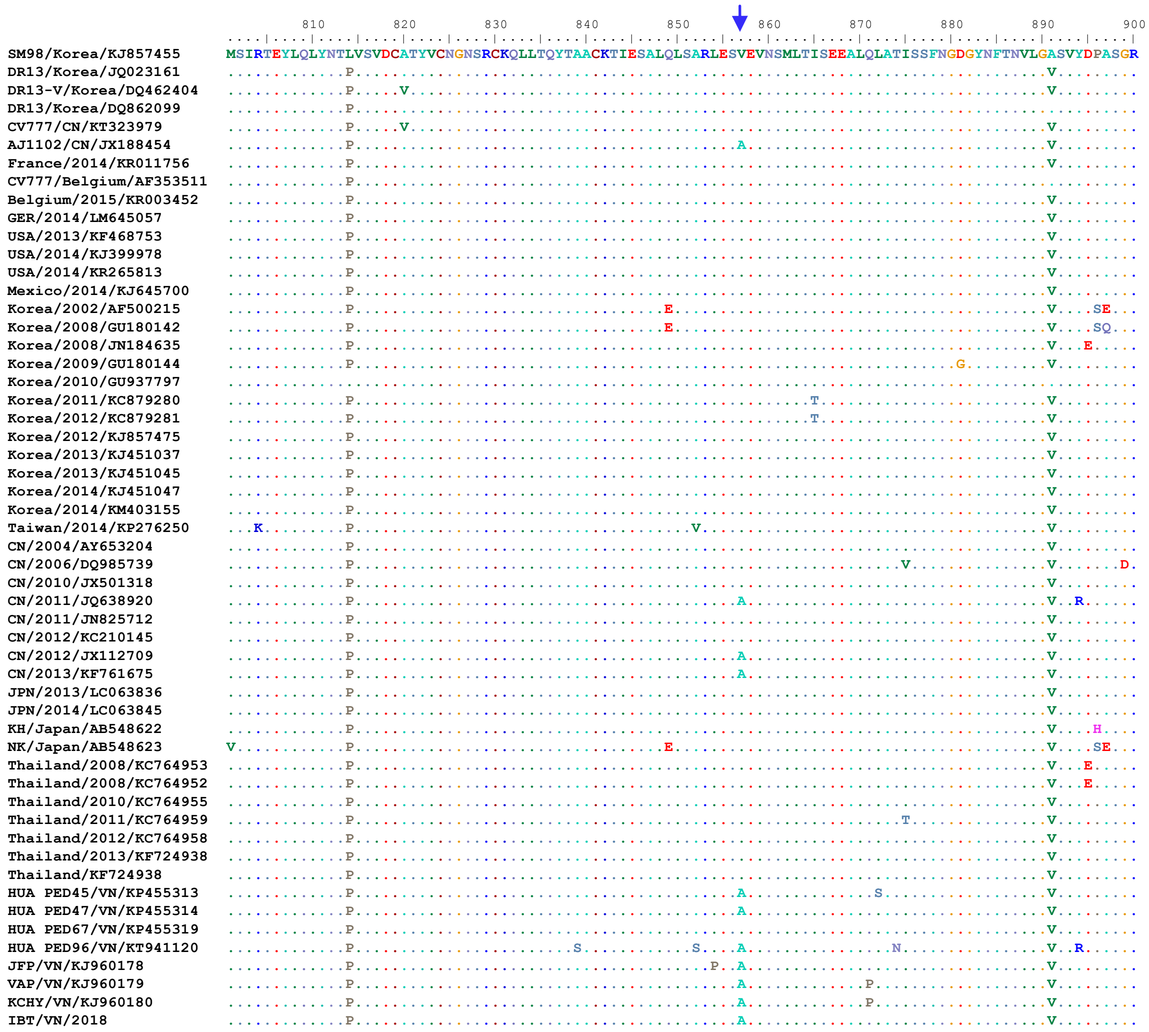

810 820 830 840 850 860 870 880 890 900
SM98/Korea/KJ857455 MSIRTEYLQLYNTLVSVDCATYVCNGNSRCKQLLTQYTAACKTIESALQLSARLESVEVNSMLTISEEALQLATISSFNGDGYNFTNVLGASVYDPASGR
DR13/Korea/JQ023161
DR13-V/Korea/DQ462404
DR13/Korea/DQ862099
CV777/CN/KT323979
AJ1102/CN/JX188454
France/2014/KR011756
CV777/Belgium/AF353511
Belgium/2015/KR003452
GER/2014/LM645057
USA/2013/KF468753
USA/2014/KJ399978
USA/2014/KR265813
Mexico/2014/KJ645700
Korea/2002/AF500215
Korea/2008/GU180142
Korea/2008/JN184635
Korea/2009/GU180144
Korea/2010/GU937797
Korea/2011/KC879280
Korea/2012/KC879281
Korea/2012/KJ857475
Korea/2013/KJ451037
Korea/2013/KJ451045
Korea/2014/KJ451047
Korea/2014/KM403155
Taiwan/2014/KP276250
CN/2004/AY653204
CN/2006/DQ985739
CN/2010/JX501318
CN/2011/JQ638920
CN/2011/JN825712
CN/2012/KC210145
CN/2012/JX112709
CN/2013/KF761675
JPN/2013/LC063836
JPN/2014/LC063845
KH/Japan/AB548622
NK/Japan/AB548623
Thailand/2008/KC764953
Thailand/2008/KC764952
Thailand/2010/KC764955
Thailand/2011/KC764959
Thailand/2012/KC764958
Thailand/2013/KF724938
Thailand/KF724938
HUA PED45/VN/KP455313
HUA PED47/VN/KP455314
HUA PED67/VN/KP455319
HUA PED96/VN/KT941120
JFP/VN/KJ960178
VAP/VN/KJ960179
KCHY/VN/KJ960180
IBT/VN/2018

```
                                   910       920       930       940       950       960       970       980       990      1000
                           ....|....|....|....|....|....|....|....|....|....|....|....|....|....|....|....|....|....|....|....|
SM98/Korea/KJ857455        VVQKRSVIEDLLFNKVVTNGLGTVDEDYKRCSNGRSVADLVCAQYYSGVMVLPGVVDAEKLHMYSASLIGGMALGGITAAAALPFSYAVQARLNYLALQT
DR13/Korea/JQ023161        ......F.............................................................................................
DR13-V/Korea/DQ462404      ....G.F.....................................................................L.S....S..H.............
DR13/Korea/DQ862099        ....................................................................................................
CV777/CN/KT323979          ....G.F.....................................................................L.T.......H.............
AJ1102/CN/JX188454         ......F.................................................................V...F.......................
France/2014/KR011756       ......F.................................................................V...F.S.....................
CV777/Belgium/AF353511     ....................................................................................................
Belgium/2015/KR003452      ......F.................................................................V...F.S.....................
GER/2014/LM645057          ......F.................................................................V...F.S.....................
USA/2013/KF468753          ......F.................................................................V...F.S.....................
USA/2014/KJ399978          ......F.................................................................V...F.S.....................
USA/2014/KR265813          ......F.................................................................V...F.S.....................
Mexico/2014/KJ645700       ......F.................................................................V...F.S.....................
Korea/2002/AF500215        ..HE..F.........X................................I...........................F...V...................
Korea/2008/GU180142        .IHE..F..........................................I...........................F...V...................
Korea/2008/JN184635        ..H...F.....................................................................F.......................
Korea/2009/GU180144        ......I.............................................................V...V...F.......................
Korea/2010/GU937797        ................................................................................T...................
Korea/2011/KC879280        ..H...F..G........................L......................P..............V...F.......................
Korea/2012/KC879281        ..H...F...........................L.....................................V...F.......................
Korea/2012/KJ857475        ......F.................................................................V...F.S.........I...........
Korea/2013/KJ451037        ......F.................................................................V...F.S.....................
Korea/2013/KJ451045        ..H...F.....................................................................F.S.....................
Korea/2014/KJ451047        ......F.................................................................V...F.S.....................
Korea/2014/KM403155        ......F.................................................................V...F.S.....................
Taiwan/2014/KP276250       ......F.................................................................V...F.S.....................
CN/2004/AY653204           ..H...F.....................................................................F.V.....................
CN/2006/DQ985739           ......F............................P....................................V...F.................EI....
CN/2010/JX501318           ......F.................................................................V...F.S.....................
CN/2011/JQ638920           ......F..E..............................................................V...F.S.....................
CN/2011/JN825712           ......F.................................................................V...F.S.....................
CN/2012/KC210145           ......F.................................................................V...F.S.....................
CN/2012/JX112709           ......F.................................................................V...F.......................
CN/2013/KF761675           ......F.................................................................V...F.S.....................
JPN/2013/LC063836          ......F.................................................................V...F.S.....................
JPN/2014/LC063845          ......F.................................................................V...F.S.....................
KH/Japan/AB548622          ..H...F.....................................................................F.......................
NK/Japan/AB548623          .IH...F..........................................I..........................F...V...................
Thailand/2008/KC764953     ..H...F.....................................................................F...................V...
Thailand/2008/KC764952     ..H...F.....................................................................F.......................
Thailand/2010/KC764955     ......F............................A....................................VF..F.......................
Thailand/2011/KC764959     ......F............................A....................................VF..F.......................
Thailand/2012/KC764958     ......F................A...........A....................................VF..F.......................
Thailand/2013/KF724938     ....K.F.........I..................A....................................VF..F.......................
Thailand/KF724938          ......F............................A....................................VF..F.......................
HUA PED45/VN/KP455313      ......F.................................................................V...F.......................
HUA PED47/VN/KP455314      ......F.................................................................V...F.......................
HUA PED67/VN/KP455319      ......F.................................................................V...F.......................
HUA PED96/VN/KT941120      ......F.................................................................V...F.S.....................
JFP/VN/KJ960178            ......F.................................................................V...F.......................
VAP/VN/KJ960179            ......F.................................................................V...F.......................
KCHY/VN/KJ960180           ......F.................................................................V...F.......................
IBT/VN/2018                ......F.................................................................V...F.S.....................
```

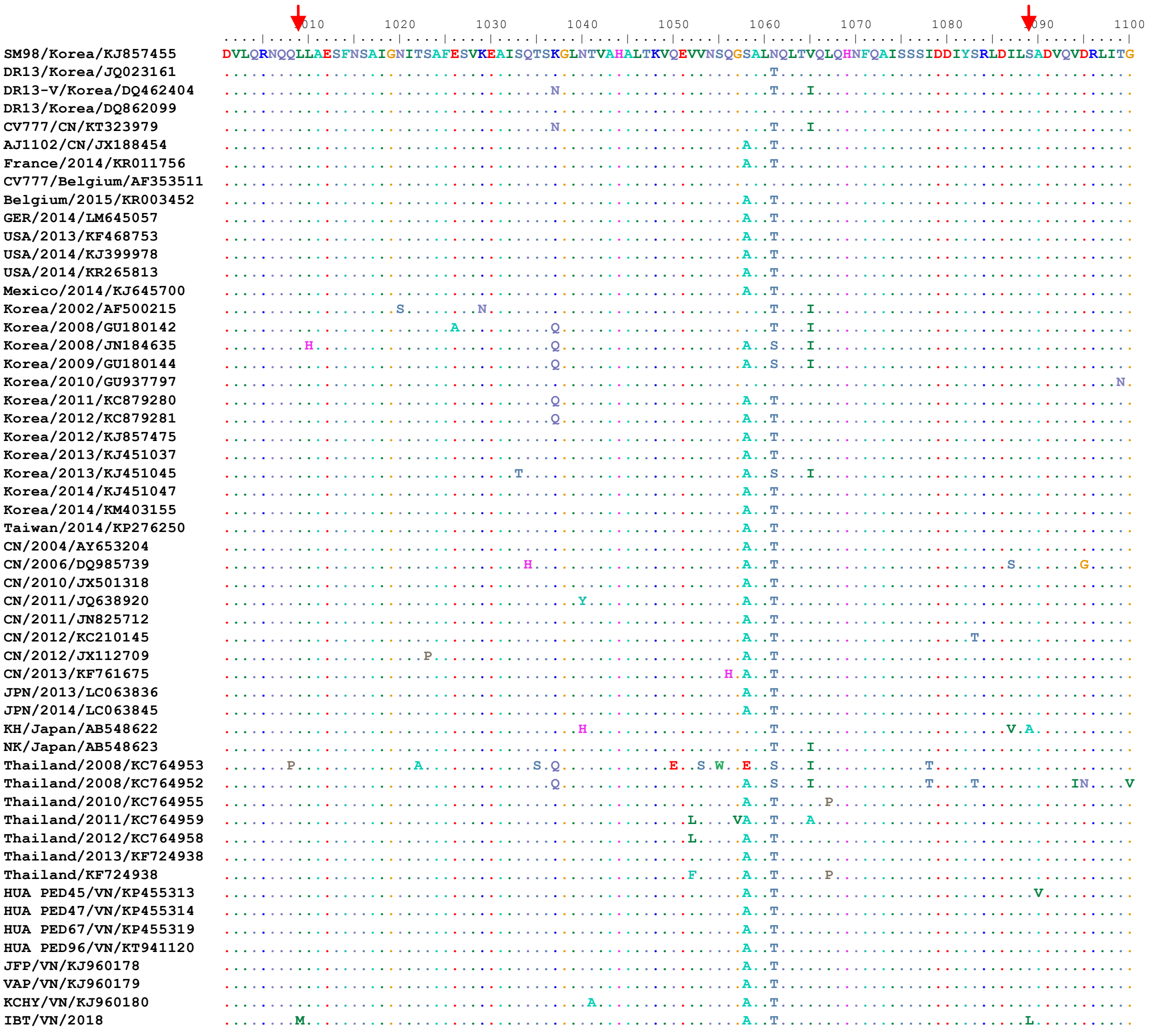
1010
1020
1030
1040
1050
1060
1070
1080
1090
1100
SM98/Korea/KJ857455
DVLQRNQQLLAESFNSAIGNITSAFESVKEAISQTSKGLNTVAHALTKVQEVVNSQGSALNQLTVQLQHNFQAISSSIDDIYSRLDILSADVQVDRLITG
DR13/Korea/JQ023161
DR13-V/Korea/DQ462404
DR13/Korea/DQ862099
CV777/CN/KT323979
AJ1102/CN/JX188454
France/2014/KR011756
CV777/Belgium/AF353511
Belgium/2015/KR003452
GER/2014/LM645057
USA/2013/KF468753
USA/2014/KJ399978
USA/2014/KR265813
Mexico/2014/KJ645700
Korea/2002/AF500215
Korea/2008/GU180142
Korea/2008/JN184635
Korea/2009/GU180144
Korea/2010/GU937797
Korea/2011/KC879280
Korea/2012/KC879281
Korea/2012/KJ857475
Korea/2013/KJ451037
Korea/2013/KJ451045
Korea/2014/KJ451047
Korea/2014/KM403155
Taiwan/2014/KP276250
CN/2004/AY653204
CN/2006/DQ985739
CN/2010/JX501318
CN/2011/JQ638920
CN/2011/JN825712
CN/2012/KC210145
CN/2012/JX112709
CN/2013/KF761675
JPN/2013/LC063836
JPN/2014/LC063845
KH/Japan/AB548622
NK/Japan/AB548623
Thailand/2008/KC764953
Thailand/2008/KC764952
Thailand/2010/KC764955
Thailand/2011/KC764959
Thailand/2012/KC764958
Thailand/2013/KF724938
Thailand/KF724938
HUA PED45/VN/KP455313
HUA PED47/VN/KP455314
HUA PED67/VN/KP455319
HUA PED96/VN/KT941120
JFP/VN/KJ960178
VAP/VN/KJ960179
KCHY/VN/KJ960180
IBT/VN/2018

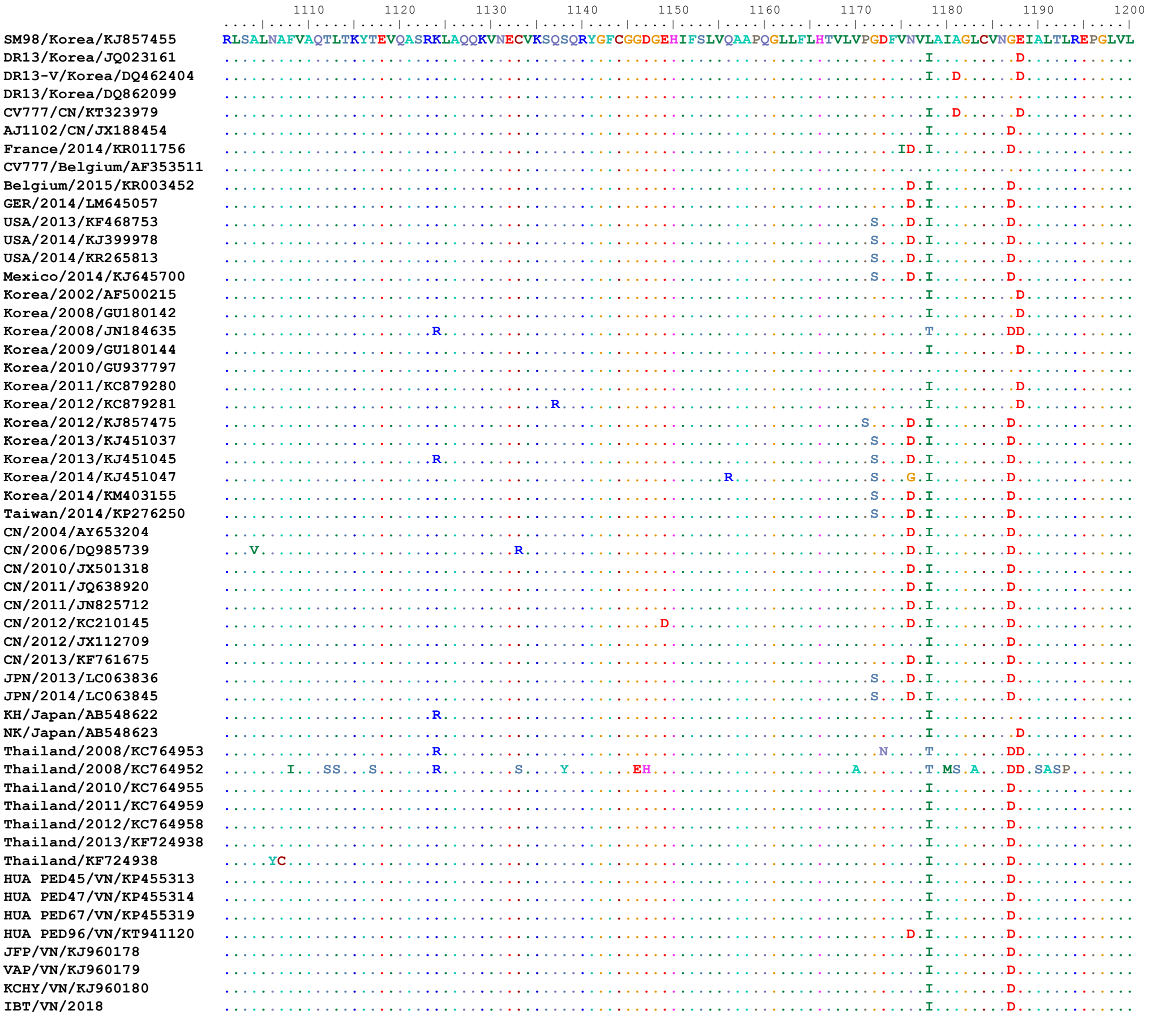
1110 1120 1130 1140 1150 1160 1170 1180 1190 1200
SM98/Korea/KJ857455 RLSALNAFVAQTLTKYTEVQASRKLAQQKVNECVKSQSQRYGFCGGDGEHIFSLVQAAPQGLLFLHTVLVPGDFVNVLAIAGLCVNGEIALTLREPGLVL
DR13/Korea/JQ023161
DR13-V/Korea/DQ462404
DR13/Korea/DQ862099
CV777/CN/KT323979
AJ1102/CN/JX188454
France/2014/KR011756
CV777/Belgium/AF353511
Belgium/2015/KR003452
GER/2014/LM645057
USA/2013/KF468753
USA/2014/KJ399978
USA/2014/KR265813
Mexico/2014/KJ645700
Korea/2002/AF500215
Korea/2008/GU180142
Korea/2008/JN184635
Korea/2009/GU180144
Korea/2010/GU937797
Korea/2011/KC879280
Korea/2012/KC879281
Korea/2012/KJ857475
Korea/2013/KJ451037
Korea/2013/KJ451045
Korea/2014/KJ451047
Korea/2014/KM403155
Taiwan/2014/KP276250
CN/2004/AY653204
CN/2006/DQ985739
CN/2010/JX501318
CN/2011/JQ638920
CN/2011/JN825712
CN/2012/KC210145
CN/2012/JX112709
CN/2013/KF761675
JPN/2013/LC063836
JPN/2014/LC063845
KH/Japan/AB548622
NK/Japan/AB548623
Thailand/2008/KC764953
Thailand/2008/KC764952
Thailand/2010/KC764955
Thailand/2011/KC764959
Thailand/2012/KC764958
Thailand/2013/KF724938
Thailand/KF724938
HUA PED45/VN/KP455313
HUA PED47/VN/KP455314
HUA PED67/VN/KP455319
HUA PED96/VN/KT941120
JFP/VN/KJ960178
VAP/VN/KJ960179
KCHY/VN/KJ960180
IBT/VN/2018

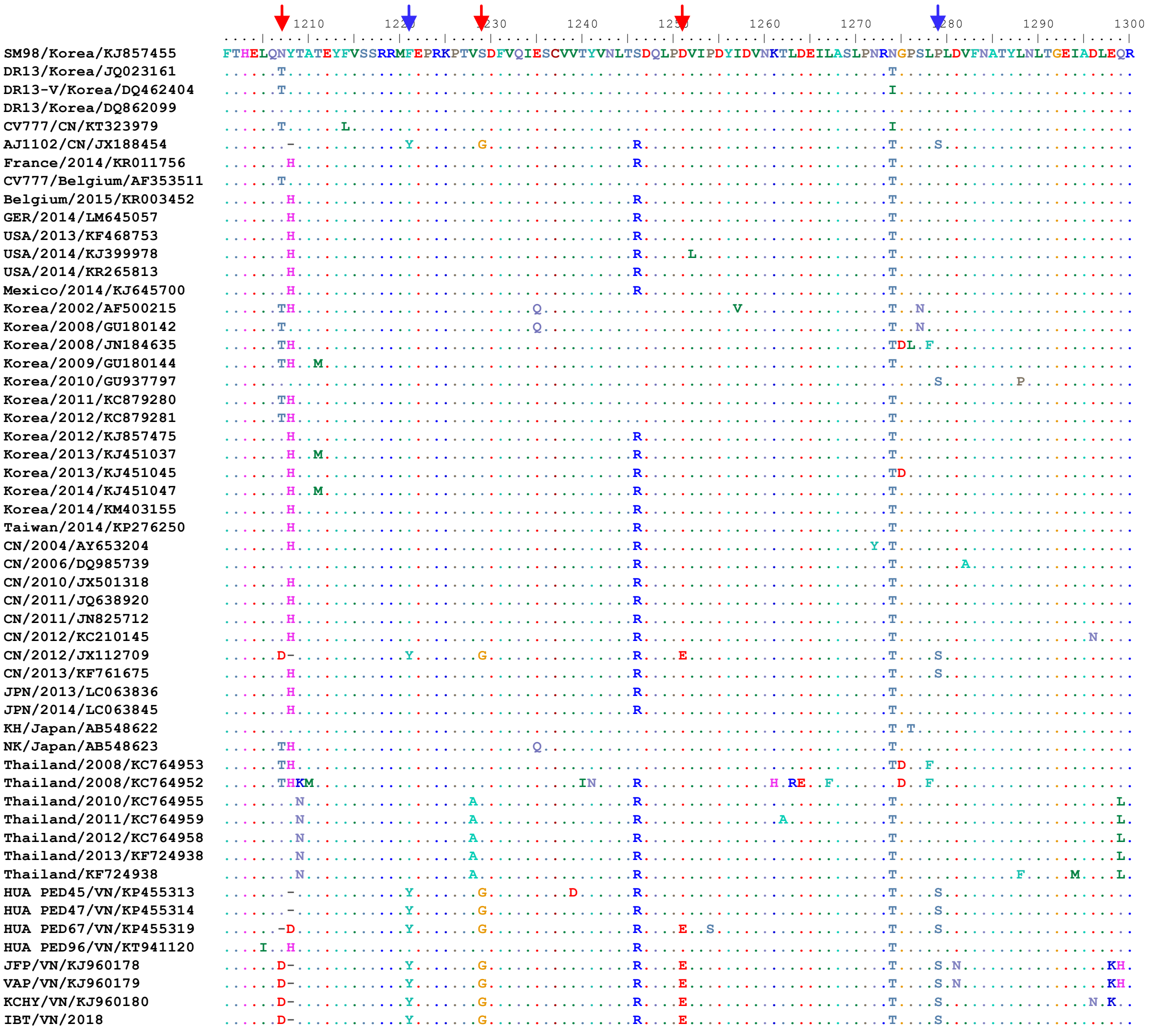
1210
1220
1230
1240
1250
1260
1270
1280
1290
1300
SM98/Korea/KJ857455
FTHELQNYTATEYFVSSRRMFEPRKPTVSDFVQIESCVVTYVNLTSDQLPDVIPDYIDVNKTLDEILASLPNRNGPSLPLDVFNATYLNLTGEIADLEQR
DR13/Korea/JQ023161
DR13-V/Korea/DQ462404
DR13/Korea/DQ862099
CV777/CN/KT323979
AJ1102/CN/JX188454
France/2014/KR011756
CV777/Belgium/AF353511
Belgium/2015/KR003452
GER/2014/LM645057
USA/2013/KF468753
USA/2014/KJ399978
USA/2014/KR265813
Mexico/2014/KJ645700
Korea/2002/AF500215
Korea/2008/GU180142
Korea/2008/JN184635
Korea/2009/GU180144
Korea/2010/GU937797
Korea/2011/KC879280
Korea/2012/KC879281
Korea/2012/KJ857475
Korea/2013/KJ451037
Korea/2013/KJ451045
Korea/2014/KJ451047
Korea/2014/KM403155
Taiwan/2014/KP276250
CN/2004/AY653204
CN/2006/DQ985739
CN/2010/JX501318
CN/2011/JQ638920
CN/2011/JN825712
CN/2012/KC210145
CN/2012/JX112709
CN/2013/KF761675
JPN/2013/LC063836
JPN/2014/LC063845
KH/Japan/AB548622
NK/Japan/AB548623
Thailand/2008/KC764953
Thailand/2008/KC764952
Thailand/2010/KC764955
Thailand/2011/KC764959
Thailand/2012/KC764958
Thailand/2013/KF724938
Thailand/KF724938
HUA PED45/VN/KP455313
HUA PED47/VN/KP455314
HUA PED67/VN/KP455319
HUA PED96/VN/KT941120
JFP/VN/KJ960178
VAP/VN/KJ960179
KCHY/VN/KJ960180
IBT/VN/2018

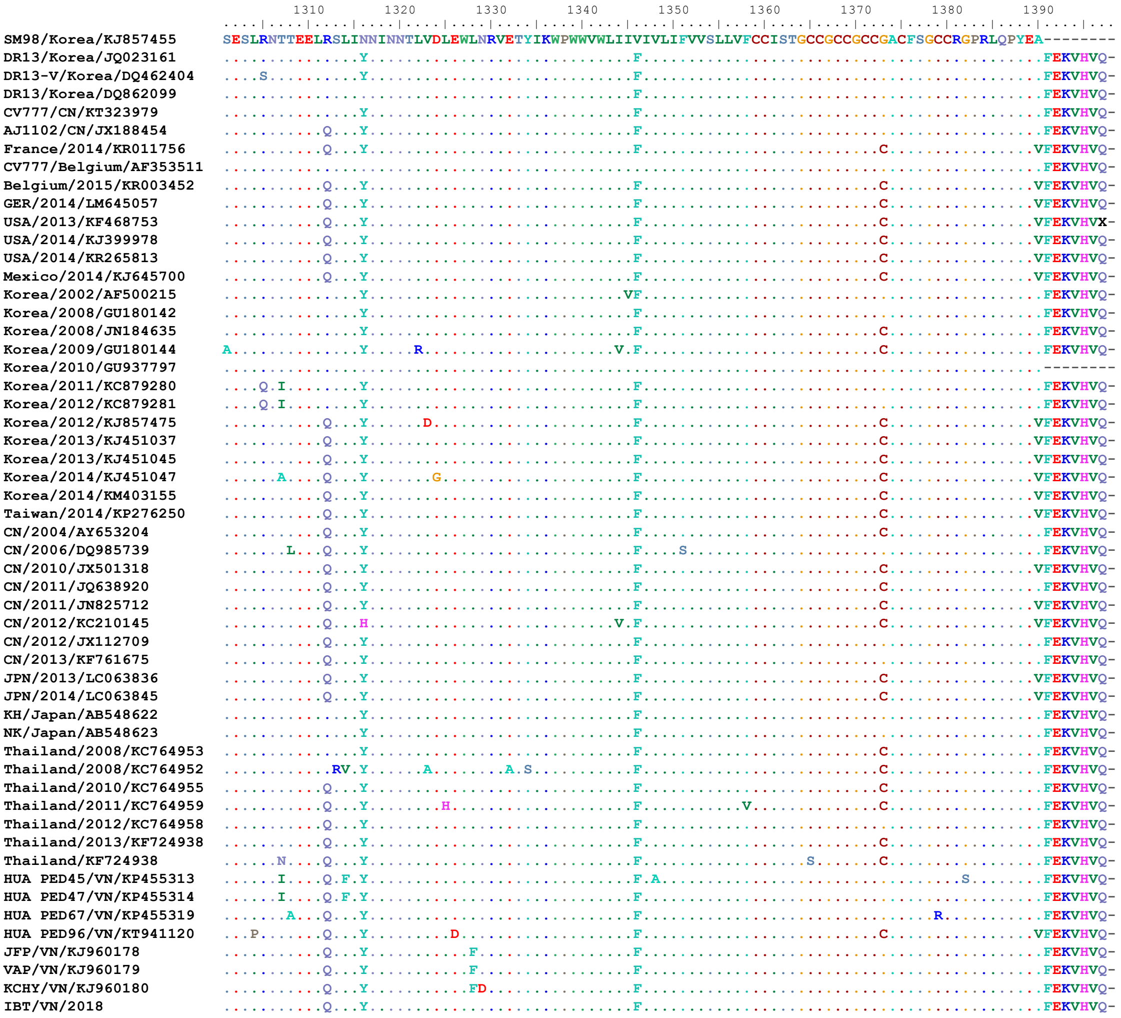

Figure S1. Comparison of amino acid sequences of S gene. The blue arrow pointed the insertions (at aa positions ^{59}NQGV^{62} and ^{145}N), deletion (at aa position ^{168}DI^{169}), and the substitutions at aa ^{135}DN^{136} > ^{135}SI^{136}, ^{497}R > T^{497}, ^{506}I > T^{506}, ^{857}V > A^{857}, ^{1221}F > Y^{1221}, and ^{1279}P > S^{1279} in S protein of IBT/VN/2018 strain when comparison with the vaccine strains CV777/CN, SM98/Korea and DR13/Korea. The red arrow pointed the substitutions at aa ^{144}N > D^{144}, ^{294}I > M^{294}, ^{318}A > S^{318}, ^{335}V > I^{335}, ^{361}A > T^{361}, ^{501}SH^{502} > ^{501}IY^{502}, ^{682}L > F^{682}, ^{777}P > L^{777}, ^{1009}L > M^{1009}, ^{1089}S > L^{1089}, ^{1207}T > D^{1207}, ^{1229}S > G^{1229}, and ^{1251}D > E^{1251} in S protein of IBT/VN/2018 strain when comparison with the vaccine strains AJ1102/CN, CV777/CN, SM98/Korea and DR13/Korea.
