# Supplementary material for: Changes in the spike and nucleocapsid protein of porcine epidemic diarrhea virus strain in Vietnam—a molecular potential for the vaccine development?
Source: PeerJ. 2021 Oct 18;9:e12329. doi: 10.7717/peerj.12329 (PMC8530102; doi:10.7717/peerj.12329)
Supplement: Supplemental Information 2 — The blue arrow pointed the changes at aa positions 216V > M216, 400E > D400 in PEDV-VN strains (IBT/VN/2018, KCHY, VAP, and JFP) and some of PEDV strains (from China and Thailand) compared to the vaccine strains CV777/CN and DR13/Korea. The green arrow pointed the changes at aa positions 364V > I364 and 378N > S378 between IBT/VN/2018 strain and other strains. [file peerj-09-12329-s002.pdf]

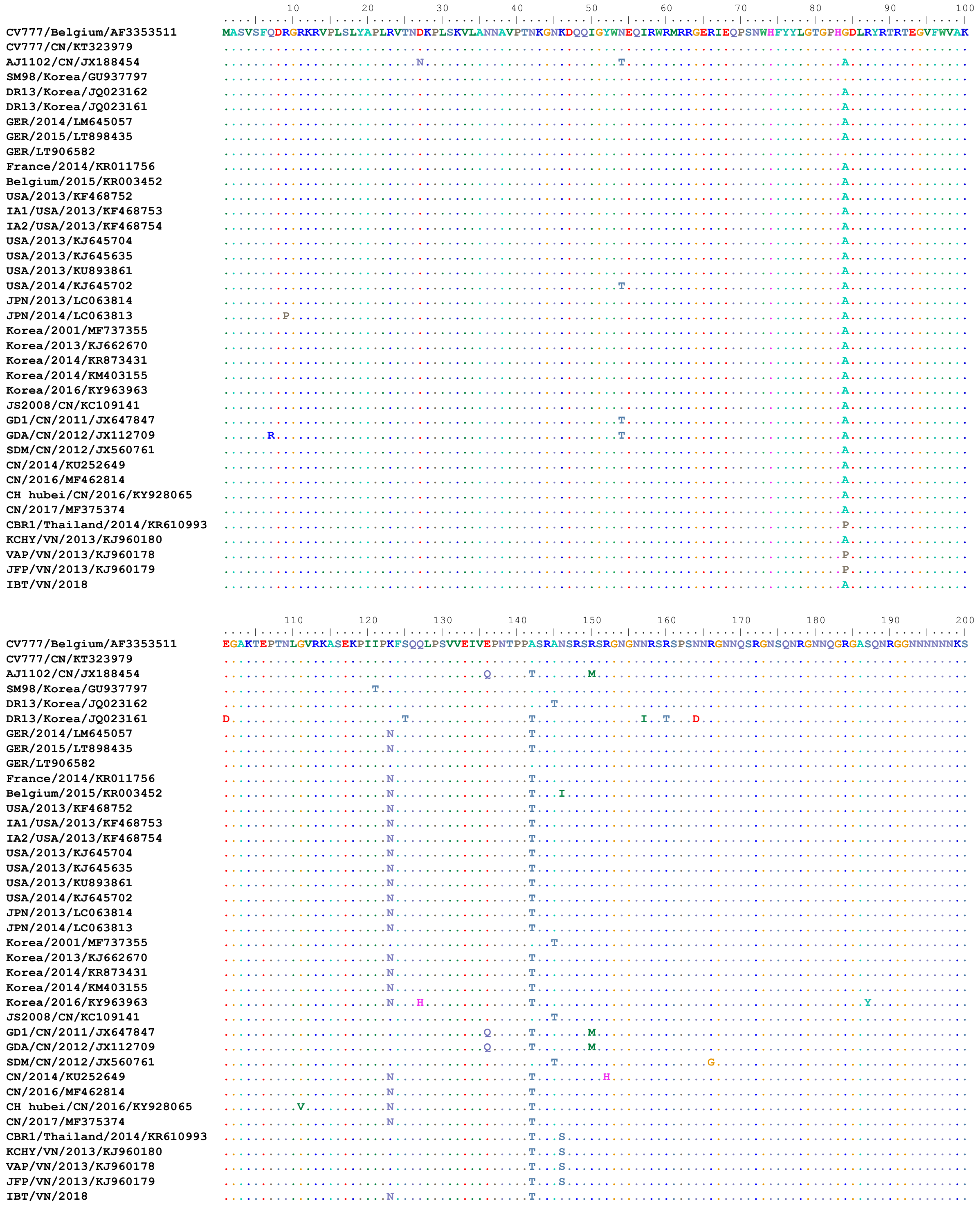
10 20 30 40 50 60 70 80 90 100
CV777/Belgium/AF3353511 MASVSFQDRGRKRVPLSLYAPLRVTNDKPLSKVLANNAVPTNKGNKDQQIGYWNEQIRWRMRRGERIEQPSNWHFYYLGTGPHGDLRYRTRTEGVFWVAK
110 120 130 140 150 160 170 180 190 200
CV777/Belgium/AF3353511 EGAKTEPTNLGVRKASEKPIIPKFSQQLPSVVEIVEPNTPPASRANSRSRSRGNGNNRSRSPSNNRGNNQSRGNSQNRGNNQGRGASQNRGGNNNNNNKS
CV777/CN/KT323979
AJ1102/CN/JX188454
SM98/Korea/GU937797
DR13/Korea/JQ023162
DR13/Korea/JQ023161
GER/2014/LM645057
GER/2015/LT898435
GER/LT906582
France/2014/KR011756
Belgium/2015/KR003452
USA/2013/KF468752
IA1/USA/2013/KF468753
IA2/USA/2013/KF468754
USA/2013/KJ645704
USA/2013/KJ645635
USA/2013/KU893861
USA/2014/KJ645702
JPN/2013/LC063814
JPN/2014/LC063813
Korea/2001/MF737355
Korea/2013/KJ662670
Korea/2014/KR873431
Korea/2014/KM403155
Korea/2016/KY963963
JS2008/CN/KC109141
GD1/CN/2011/JX647847
GDA/CN/2012/JX112709
SDM/CN/2012/JX560761
CN/2014/KU252649
CN/2016/MF462814
CH hubei/CN/2016/KY928065
CN/2017/MF375374
CBR1/Thailand/2014/KR610993
KCHY/VN/2013/KJ960180
VAP/VN/2013/KJ960178
JFP/VN/2013/KJ960179
IBT/VN/2018

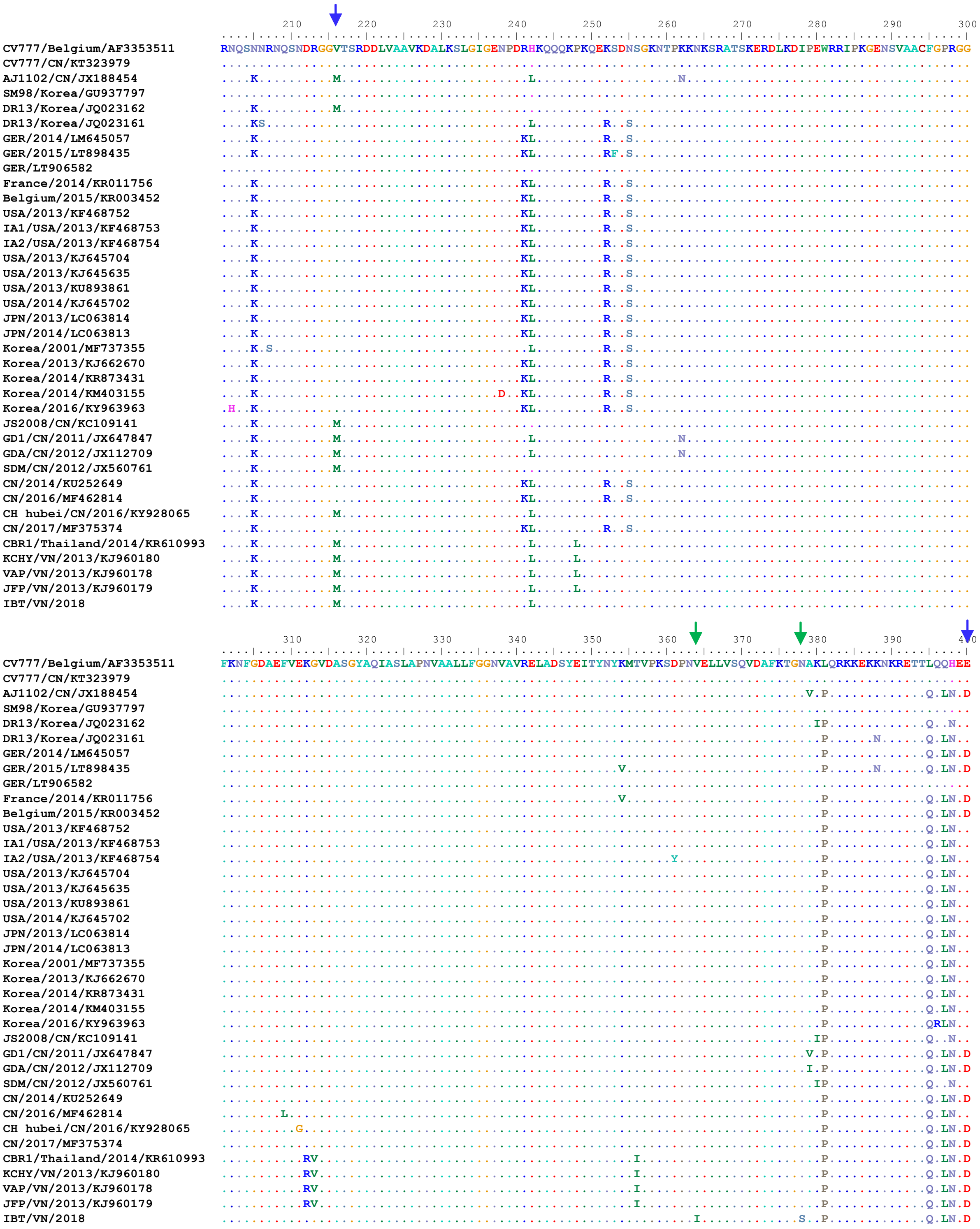

210 220 230 240 250 260 270 280 290 300
CV777/Belgium/AF3353511
RNQSNNRNQSNDRGGVTSRDDLVAAVKDALKSLGIGENPDRHKQQQKPKQEKSDNSGKNTPKKNKSRATSKERDLKDIPEWRRIPKGENSVAACFGPRGG
CV777/CN/KT323979
AJ1102/CN/JX188454
SM98/Korea/GU937797
DR13/Korea/JQ023162
DR13/Korea/JQ023161
GER/2014/LM645057
GER/2015/LT898435
GER/LT906582
France/2014/KR011756
Belgium/2015/KR003452
USA/2013/KF468752
IA1/USA/2013/KF468753
IA2/USA/2013/KF468754
USA/2013/KJ645704
USA/2013/KJ645635
USA/2013/KU893861
USA/2014/KJ645702
JPN/2013/LC063814
JPN/2014/LC063813
Korea/2001/MF737355
Korea/2013/KJ662670
Korea/2014/KR873431
Korea/2014/KM403155
Korea/2016/KY963963
JS2008/CN/KC109141
GD1/CN/2011/JX647847
GDA/CN/2012/JX112709
SDM/CN/2012/JX560761
CN/2014/KU252649
CN/2016/MF462814
CH hubei/CN/2016/KY928065
CN/2017/MF375374
CBR1/Thailand/2014/KR610993
KCHY/VN/2013/KJ960180
VAP/VN/2013/KJ960178
JFP/VN/2013/KJ960179
IBT/VN/2018
310 320 330 340 350 360 370 380 390 400
FKNFGDAEFVEKGVDASGYAQIASLAPNVAALLFGGNVAVRELADSYEITYNYKMTVPKSDPNVELLVSQVDAFKTGNAKLQRKKEKKNKRETTLQQHEE

```
                                      410       420       430       440
                              ....|....|....|....|....|....|....|....|..
CV777/Belgium/AF3353511       AIYDDVGAPSDVTHANLEWDTAVDGGDTAVEIINEIFDTGN*
CV777/CN/KT323979             .........................................*
AJ1102/CN/JX188454            ......V..................................*
SM98/Korea/GU937797           .........................................*
DR13/Korea/JQ023162           ......V..................................*
DR13/Korea/JQ023161           ......V.....V............................*
GER/2014/LM645057             ......L...S..............................*
GER/2015/LT898435             ......L...S..............................*
GER/LT906582                  .........................................*
France/2014/KR011756          ......L...S..............................*
Belgium/2015/KR003452         ......L...S..............................*
USA/2013/KF468752             ......V..................................*
IA1/USA/2013/KF468753         ......V..................................*
IA2/USA/2013/KF468754         ......V..................................*
USA/2013/KJ645704             ......V..................................*
USA/2013/KJ645635             ......V..................................*
USA/2013/KU893861             ......V..................................*
USA/2014/KJ645702             ......V..................................*
JPN/2013/LC063814             ......V..................................*
JPN/2014/LC063813             ......V..................................*
Korea/2001/MF737355           ......V.....V...........................S*
Korea/2013/KJ662670           ......V..................................*
Korea/2014/KR873431           ......V..................................*
Korea/2014/KM403155           ......V..................................*
Korea/2016/KY963963           ......V..................................*
JS2008/CN/KC109141            ......V..................................*
GD1/CN/2011/JX647847          ......V..................................*
GDA/CN/2012/JX112709          ......V..................................*
SDM/CN/2012/JX560761          ......V..................................*
CN/2014/KU252649              ......L...S..............................*
CN/2016/MF462814              ......V..................................*
CH hubei/CN/2016/KY928065     ......VS.................................*
CN/2017/MF375374              ......LS..S..............................*
CBR1/Thailand/2014/KR610993   ......V..................................*
KCHY/VN/2013/KJ960180         ......V..................................*
VAP/VN/2013/KJ960178          ......V..................................*
JFP/VN/2013/KJ960179          ......V..................................*
IBT/VN/2018                   ......V..................................*
```

Figure S2. Comparison of amino acid sequences of N gene. The red arrow pointed the changes at aa positions $^{216}V > M^{216}$, $^{400}E > D^{400}$ in PEDV-VN strains (IBT/VN/2018, KCHY, VAP, and JFP) and some of PEDV strains (from China and Thailand) compared to CV777/AF353511 strain. The green arrow pointed the changes at aa positions $^{364}V > I^{364}$ and $^{378}N > S^{378}$ between IBT/VN/2018 strain and other strains.
