# Supplementary material for: Changes in the spike and nucleocapsid protein of porcine epidemic diarrhea virus strain in Vietnam—a molecular potential for the vaccine development?
Source: PeerJ. 2021 Oct 18;9:e12329. doi: 10.7717/peerj.12329 (PMC8530102; doi:10.7717/peerj.12329)
Supplement: Supplemental Information 3 — There were four substitutions at aa positions 25L > S25, 70I > V70, 107C > F107, and 168D > N168 that found in PEDV-VN (IBT/VN/2018, KCHY, VAP, and JFP) strains and GD1/CN/2011/JX647847, GDA/CN/2012/JX112709, CH Hubei/CN/2016/KY928065, and CBR1/Thailand/2014/KR610993 strains compared to other strains. [file peerj-09-12329-s003.pdf]

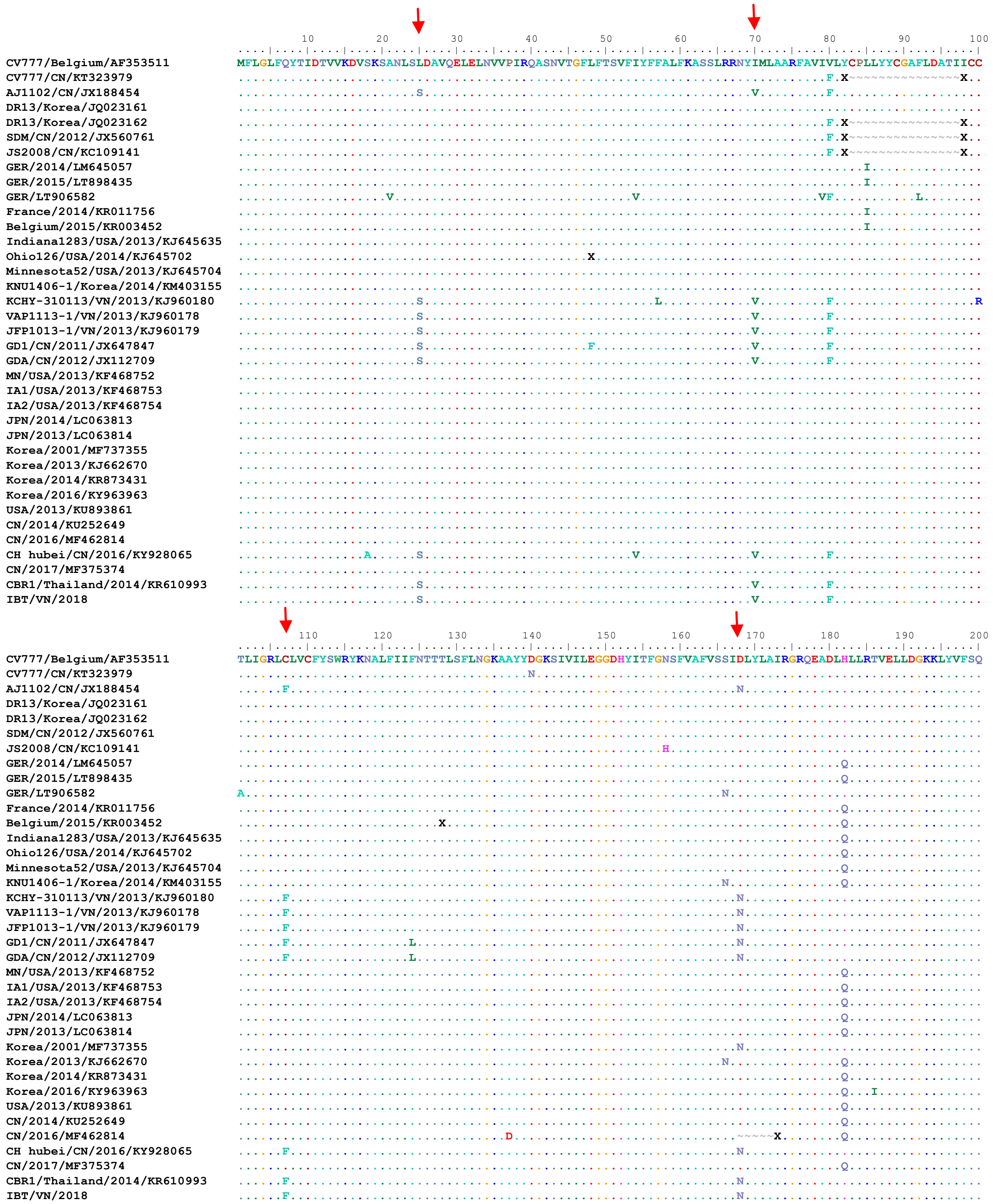

CV777/Belgium/AF353511
CV777/CN/KT323979
AJ1102/CN/JX188454
DR13/Korea/JQ023161
DR13/Korea/JQ023162
SDM/CN/2012/JX560761
JS2008/CN/KC109141
GER/2014/LM645057
GER/2015/LT898435
GER/LT906582
France/2014/KR011756
Belgium/2015/KR003452
Indiana1283/USA/2013/KJ645635
Ohio126/USA/2014/KJ645702
Minnesota52/USA/2013/KJ645704
KNU1406-1/Korea/2014/KM403155
KCHY-310113/VN/2013/KJ960180
VAP1113-1/VN/2013/KJ960178
JFP1013-1/VN/2013/KJ960179
GD1/CN/2011/JX647847
GDA/CN/2012/JX112709
MN/USA/2013/KF468752
IA1/USA/2013/KF468753
IA2/USA/2013/KF468754
JPN/2014/LC063813
JPN/2013/LC063814
Korea/2001/MF737355
Korea/2013/KJ662670
Korea/2014/KR873431
Korea/2016/KY963963
USA/2013/KU893861
CN/2014/KU252649
CN/2016/MF462814
CH hubei/CN/2016/KY928065
CN/2017/MF375374
CBR1/Thailand/2014/KR610993
IBT/VN/2018
MFLGLFQYTIDTVVKDVSKSANLSLDAVQELELNVVPIRQASNVTGFLFTSVFIYFFALFKASSLRRNYIMLAARFAVIVLYCPLLYYCGAFLDATIICC
TLIGRLCLVCFYSWRYKNALFIIFNTTTLSFLNGKAAYYDGKSIVILEGGDHYITFGNSFVAFVSSIDLYLAIRGRQEADLHLLRTVELLDGKKLYVFSQ

```
                                          210       220
                                 ....|....|....|....|....|
CV777/Belgium/AF353511           HQIVGITNAAFDSIQLDEYATISE*
CV777/CN/KT323979                ........................*
AJ1102/CN/JX188454               ........................*
DR13/Korea/JQ023161              ........................*
DR13/Korea/JQ023162              ........................*
SDM/CN/2012/JX560761             ........................*
JS2008/CN/KC109141               ........................*
GER/2014/LM645057                ........................*
GER/2015/LT898435                ........................*
GER/LT906582                     ........................*
France/2014/KR011756             ........................*
Belgium/2015/KR003452            ........................*
Indiana1283/USA/2013/KJ645635    ........................*
Ohio126/USA/2014/KJ645702        ........................*
Minnesota52/USA/2013/KJ645704    ........................*
KNU1406-1/Korea/2014/KM403155    ........................*
KCHY-310113/VN/2013/KJ960180     ........................*
VAP1113-1/VN/2013/KJ960178       ..V.....................*
JFP1013-1/VN/2013/KJ960179       ..V.....................*
GD1/CN/2011/JX647847             ........................*
GDA/CN/2012/JX112709             .H......................*
MN/USA/2013/KF468752             ........................*
IA1/USA/2013/KF468753            ........................*
IA2/USA/2013/KF468754            ........................*
JPN/2014/LC063813                ........................*
JPN/2013/LC063814                ........................*
Korea/2001/MF737355              ........................*
Korea/2013/KJ662670              ........................*
Korea/2014/KR873431              ........................*
Korea/2016/KY963963              ........................*
USA/2013/KU893861                ........................*
CN/2014/KU252649                 ........................*
CN/2016/MF462814                 ........................*
CH hubei/CN/2016/KY928065        ........................*
CN/2017/MF375374                 ........................*
CBR1/Thailand/2014/KR610993      ..V.....................*
IBT/VN/2018                      ........................*
```

Figure S3. Comparison of amino acid sequences of ORF3 gene. There were four substitutions at aa positions $^{25}L > S^{25}$, $^{70}I > V^{70}$, $^{107}C > F^{107}$, and $^{168}D > N^{168}$ that found in PEDV-VN (IBT/VN/2018, KCHY, VAP, and JFP) strains and GD1/CN/2011/JX647847, GDA/CN/2012/JX112709, CH Hubei/CN/2016/KY928065, and CBR1/Thailand/2014/KR610993 strains compared to other strains.
