# Supplementary material for: Changes in the spike and nucleocapsid protein of porcine epidemic diarrhea virus strain in Vietnam—a molecular potential for the vaccine development?
Source: PeerJ. 2021 Oct 18;9:e12329. doi: 10.7717/peerj.12329 (PMC8530102; doi:10.7717/peerj.12329)
Supplement: Supplemental Information 4 — No changes were observed in the 3D structure of the S protein of IBT/VN/2018 at mutated sites p.P766L, p.L1009M, and p.S1089L as compared to 6U7K strain. [file peerj-09-12329-s004.pdf]

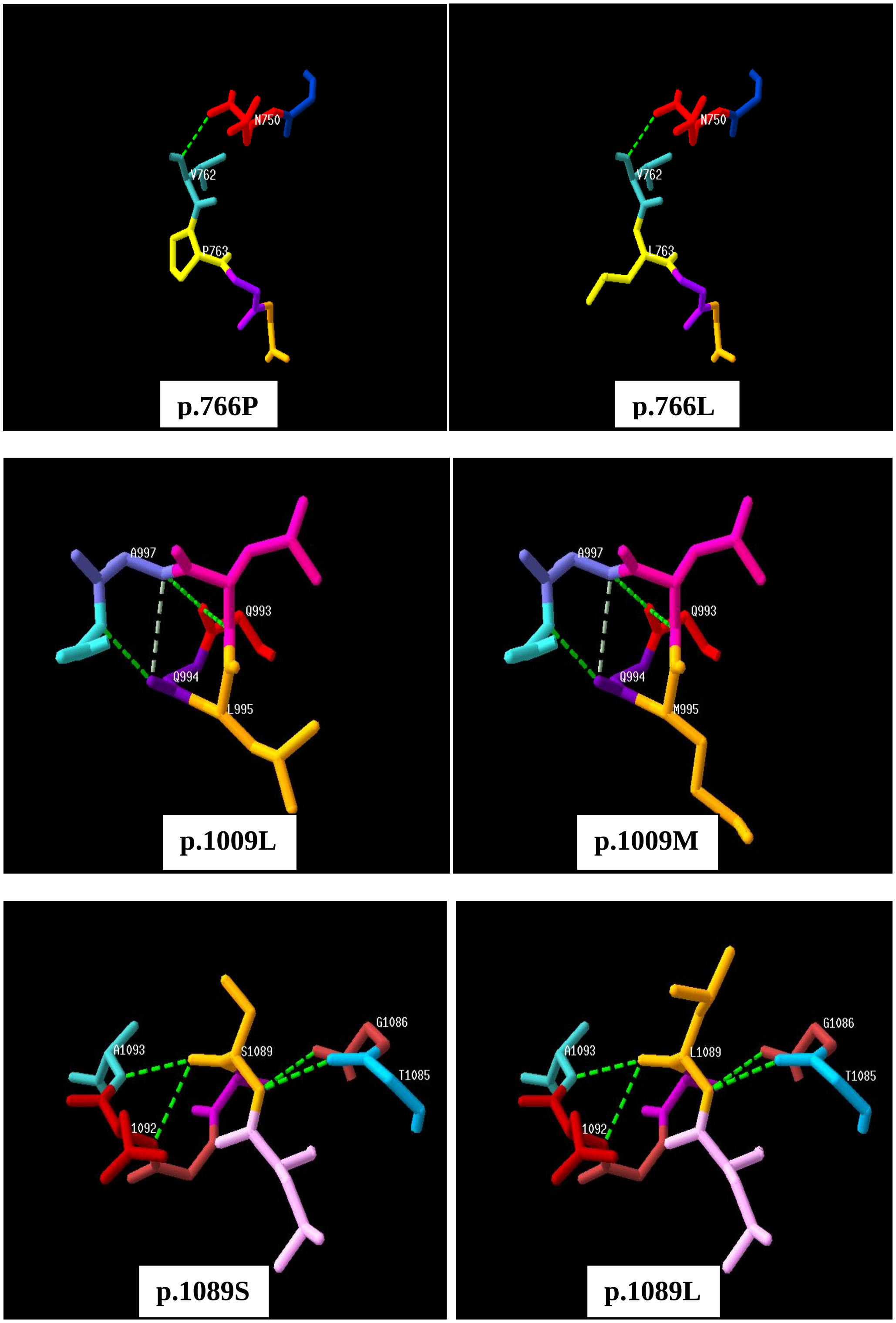

Figure S4. Homology modeling predicts the 3D structure of S protein of IBT/VN/2018 through sequence alignment of the known 3D structure strain (6U7K). No changes were observed in the 3D structure of the S protein of IBT/VN/2018 at mutated sites p.P766L, p.L1009M, and p.S1089L as compared to 6U7K strain.
