# Supplementary material for: Changes in the spike and nucleocapsid protein of porcine epidemic diarrhea virus strain in Vietnam—a molecular potential for the vaccine development?
Source: PeerJ. 2021 Oct 18;9:e12329. doi: 10.7717/peerj.12329 (PMC8530102; doi:10.7717/peerj.12329)
Supplement: Supplemental Information 5 [file peerj-09-12329-s005.pdf]

Table S1: Genetic similarity of complete nucleotide sequences (%) between the IBT/VN/2018 and other reference sequences.

**Strain**	**%**	**Strain**	**%**
CV777/Belgium/AF353511	96.3	SDM/CN/2012/JX560761	96.6
CV777/China/KT323979	96.5	KCHY-310113/VN/2013/KJ960180	97.8
DR13/Korea/JQ023162	96.5	VAP1113-1/VN/2013/KJ960178	97.8
DR13/Korea/JQ023161	97.1	JFP1013-1/VN/2013/KJ960179	97.8
SM98/Korea/GU937797	95.9	JS2008/CN/KC109141	96.6
AJ1102/China/JX188454	98.1	GD1/CN/2011/JX647847	98.1
GER/2014/LM645057	97.5	GDA/CN/2012/JX112709	98.1
GER/2015/LT898435	97.4	MN/USA/2013/KF468752	97.9
GER/LT906582	96.2	IA1/USA/2013/KF468753	97.9
France/2014/KR011756	97.4	IA2/USA/2013/KF468754	97.9
Belgium/2015/KR003452	97.4	JPN/2014/LC063813	97.4
Indiana1283/USA/2013/KJ645635	97.4	JPN/2013/LC063814	97.5
Ohio126/USA/2014/KJ645702	97.4	Korea/2001/MF737355	97.5
Minnesota52/USA/2013/KJ645704	97.4	Korea/2013/KJ662670	97.9
USA/2013/KU893861	97.9	Korea/2014/KR873431	97.9
CN/2014/KU252649	97.9	Korea/2016/KY963963	97.6
CN/2016/MF462814	97.7	KNU1406-1/Korea/2014/KM403155	97.5
CN/2017/MF375374	97.7	CBR1/Thailand/2014/KR610993	95.7
CH hubei/CN/2016/KY928065	99.3		
