# Supplementary material for: Changes in the spike and nucleocapsid protein of porcine epidemic diarrhea virus strain in Vietnam—a molecular potential for the vaccine development?
Source: PeerJ. 2021 Oct 18;9:e12329. doi: 10.7717/peerj.12329 (PMC8530102; doi:10.7717/peerj.12329)
Supplement: Supplemental Information 6 [file peerj-09-12329-s006.pdf]

Table S2: Genetic similarity of nucleotide sequences and amino acid sequences for the coding region of the Spike protein (%) between the IBT/VN/2018 and other reference sequences.

Strain	Nucleotide sequence (%)	Amino acid sequence (%)
France/2014/KR011756	94.9	94.4
CV777/Belgium/AF353511	93.2	92.5
Belgium/2015/KR003452	94.7	94.1
GER/2014/LM645057	94.9	94.5
DR13/Korea/JQ023161	94.2	93.6
USA/2013/KF468753	97.5	97.8
USA/2014/KJ399978	94.8	94.3
USA/2014/KR265813	97.5	97.7
Mexico/2014/KJ645700	97.4	97.6
Korea/2002/AF500215	93.9	93.0
Korea/2008/GU180142	94.2	93.7
Korea/2008/JN184635	96.1	95.2
Korea/2009/GU180144	94.5	94.1
Korea/2010/GU937797	92.2	91.1
Korea/2011/KC879280	95.9	96.2
Korea/2012/KC879281	95.9	96.1
Korea/2012/KJ857475	97.3	97.1
Korea/2013/KJ451037	95.8	94.8
Korea/2013/KJ451045	96.8	97.1
Korea/2014/KJ451047	97.1	97.0
Korea/2014/KM403155	94.7	94.2
Taiwan/2014/KP276250	97.4	97.4
CN/2004/AY653204	94.7	94.0
CN/2006/DQ985739	93.7	93.5
CN/2010/JX501318	94.9	94.2
CN/2011/JQ638920	97.7	97.6
CN/2011/JN825712	97.5	97.6
CN/2012/KC210145	97.4	97.4
CN/2012/JX112709	98.1	98.4

Strain	Nucleotide sequence (%)	Amino acid sequence (%)
CN/2013/KF761675	98.0	97.9
JPN/2013/LC063836	97.5	97.6
JPN/2014/LC063845	94.8	94.3
KH/Japan/AB548622	94.0	93.5
NK/Japan/AB548623	94.1	93.4
Thailand/2008/KC764953	96.3	95.4
Thailand/2008/KC764952	95.3	93.5
Thailand/2010/KC764955	95.4	96.1
Thailand/2011/KC764959	95.0	95.3
Thailand/2012/KC764958	94.9	95.1
Thailand/2013/KF724938	96.3	96.3
Thailand/KF724938	96.0	96.1
HUA PED45/VN/KP455313	97.4	97.5
HUA PED47/VN/KP455314	97.7	98.0
HUA PED67/VN/KP455319	94.7	94.7
HUA PED96/VN/KT941120	96.9	96.0
JFP/VNN/KJ960178	97.4	97.4
VAP/VN/KJ960179	97.6	97.8
KCHY/VN/KJ960180	97.5	97.7
SM98/Korea/KJ857455	92.4	91.2
DR13/Korea/DQ862099	93.2	92.5
CV777/CN/KT323979	93.3	92.1
AJ1102/CN/JX188454	97.8	98.3
