# Supplementary material for: Changes in the spike and nucleocapsid protein of porcine epidemic diarrhea virus strain in Vietnam—a molecular potential for the vaccine development?
Source: PeerJ. 2021 Oct 18;9:e12329. doi: 10.7717/peerj.12329 (PMC8530102; doi:10.7717/peerj.12329)
Supplement: Supplemental Information 7 [file peerj-09-12329-s007.pdf]

Table S3: Genetic similarity of nucleotide sequences and amino acid sequences for the coding region of the Nucleocapsid protein (%) between the IBT/VN/2018 and other reference sequences.

Strain	Nucleotide sequence (%)	Amino acid sequence (%)
CV777/Belgium/AF3353511	95.7	96.8
DR13/Korea/JQ023161	96.3	96.5
DR13/Korea/JQ023162	96.7	96.5
SM98/Korea/GU937797	95.6	96.5
GER/2014/LM645057	96.9	98.1
GER/2015/LT898435	96.6	97.5
GER/LT906582	95.7	96.8
France/2014/KR011756	96.8	97.9
Belgium/2015/KR003452	96.9	97.9
USA/2013/KF468752	97.2	98.4
IA1/USA/2013/KF468753	97.2	98.4
IA2/USA/2013/KF468754	97.1	98.1
USA/2013/KJ645704	97.2	98.4
USA/2013/KJ645635	97.2	98.4
USA/2013/KU893861	97.2	98.4
USA/2014/KJ645702	97.0	98.1
JPN/2013/LC063814	97.2	98.4
JPN/2014/LC063813	97.1	98.1
Korea/2001/MF737355	96.3	97.2
Korea/2013/KJ662670	97.2	98.4
Korea/2014/KR873431	97.2	98.4
Korea/2014/KM403155	97.1	98.1
Korea/2016/KY963963	96.6	97.5
JS2008/CN/KC109141	96.7	97.9
GD1/CN/2011/JX647847	97.5	98.1
GDA/CN/2012/JX112709	97.3	97.9
SDM/CN/2012/JX560761	96.6	97.7
CN/2014/KU252649	96.8	97.9
CN/2016/MF462814	96.9	98.1
CN/2017/MF375374	96.6	97.9
CH hubei/CN/2016/KY928065	99.0	98.8
CBR1/Thailand/2014/KR610993	97.3	97.9
KCHY/VN/2013/KJ960180	97.4	98.1
VAP/VN/2013/KJ960178	97.3	97.9
JFP/VN/2013/KJ960179	97.3	97.9
CV777/CN/KT323979	92.5	93.4
AJ1102/CN/JX188454	97.5	97.9
