# Supplementary material for: Changes in the spike and nucleocapsid protein of porcine epidemic diarrhea virus strain in Vietnam—a molecular potential for the vaccine development?
Source: PeerJ. 2021 Oct 18;9:e12329. doi: 10.7717/peerj.12329 (PMC8530102; doi:10.7717/peerj.12329)
Supplement: Supplemental Information 8 [file peerj-09-12329-s008.pdf]

Table S4: Genetic similarity of nucleotide sequences and amino acid sequences for the coding region of the ORF3 (%) between the IBT/VN/2018 and other reference sequences.

**Strain**	**Nucleotide sequence (%)**	**Amino acid sequence (%)**
CV777/Belgium/AF3353511	96.5	97.7
DR13/Korea/JQ023161	96.5	97.7
DR13/Korea/JQ023162	89.4	90.6
GER/2014/LM645057	95.7	96.8
GER/2015/LT898435	95.5	96.8
GER/LT906582	96.1	95.5
France/2014/KR011756	95.7	96.8
Belgium/2015/KR003452	95.5	96.4
USA/2013/KF468752	95.8	97.3
IA1/USA/2013/KF468753	95.8	97.3
IA2/USA/2013/KF468754	95.8	97.3
USA/2013/KJ645704	95.8	97.3
USA/2013/KJ645635	95.8	97.3
USA/2013/KU893861	95.8	97.3
USA/2014/KJ645702	95.5	96.8
JPN/2013/LC063814	95.8	97.3
JPN/2014/LC063813	95.7	97.3
Korea/2001/MF737355	96.4	98.2
Korea/2013/KJ662670	95.7	96.8
Korea/2014/KR873431	95.8	97.3
Korea/2014/KM403155	95.7	96.8
Korea/2016/KY963963	95.7	96.8
JS2008/CN/KC109141	89.1	90.1
GD1/CN/2011/JX647847	98.2	99.1
GDA/CN/2012/JX112709	98.5	99.1
SDM/CN/2012/JX560761	89.4	90.6
CN/2014/KU252649	95.8	97.3
CN/2016/MF462814	93.7	94.6
CN/2017/MF375374	95.7	97.3
CH hubei/CN/2016/KY928065	99.4	99.1
CBR1/Thailand/2014/KR610993	98.6	96.8
KCHY/VN/2013/KJ960180	98.5	99.1
VAP/VN/2013/KJ960178	98.6	99.5
JFP/VN/2013/KJ960179	98.6	99.5
CV777/CN/KT323979	89.1	90.1
AJ1102/CN/JX188454	98.9	100.0
